# Engineering circular RNA for enhanced protein production

**DOI:** 10.1038/s41587-022-01393-0

**Published:** 2022-07-18

**Authors:** Robert Chen, Sean K. Wang, Julia A. Belk, Laura Amaya, Zhijian Li, Angel Cardenas, Brian T. Abe, Chun-Kan Chen, Paul A. Wender, Howard Y. Chang

**Affiliations:** 1 Center for Personal Dynamic Regulomes, Stanford University, Stanford, CA USA; 2 Department of Ophthalmology, Stanford University School of Medicine, Stanford, CA USA; 3 Department of Computer Science, Stanford University, Stanford, CA USA; 4 Department of Chemistry, Stanford University, Stanford, CA USA; 5 Department of Chemical and Systems Biology, Stanford University, Stanford, CA USA; 6 Howard Hughes Medical Institute, Stanford University, Stanford, CA USA

**Keywords:** RNA splicing, Gene therapy, Gene delivery, Nucleic-acid therapeutics

## Abstract

Circular RNAs (circRNAs) are stable and prevalent RNAs in eukaryotic cells that arise from back-splicing. Synthetic circRNAs and some endogenous circRNAs can encode proteins, raising the promise of circRNA as a platform for gene expression. In this study, we developed a systematic approach for rapid assembly and testing of features that affect protein production from synthetic circRNAs. To maximize circRNA translation, we optimized five elements: vector topology, 5′ and 3′ untranslated regions, internal ribosome entry sites and synthetic aptamers recruiting translation initiation machinery. Together, these design principles improve circRNA protein yields by several hundred-fold, provide increased translation over messenger RNA in vitro, provide more durable translation in vivo and are generalizable across multiple transgenes.

## Abstract (web summary)

Protein expression from circular RNAs is enhanced several hundred-fold by optimizing vector design.

## Main

Ribonucleic acid (RNA) therapeutics—spanning messenger RNAs (mRNAs), small interfering RNAs (siRNAs) and micro RNAs (miRNAs)—have expanded into a novel pillar of modern medicine, joining small molecules, biologics and cell therapeutics. Recently, mRNA vaccines have drawn attention for addressing the severe acute respiratory syndrome coronavirus 2 (SARS-CoV-2) pandemic^1,2^. The rapid pace by which mRNAs can be designed, synthesized and tested has unlocked new ways to respond to urgent and evolving medical crises. In the backdrop of the worldwide success of mRNA medicines, circularization of coding RNAs into circRNAs has garnered considerable interest as an approach to extend the duration of protein translation. Originally investigated in the context of naturally occurring back-splicing, circRNAs are single-stranded RNA molecules covalently joined head to tail^3^. Considerable advancements have been made in synthesizing and circularizing long transcripts into circRNAs^4,5^. However, the fundamental mechanisms of translation initiation for circRNAs and mRNAs differ because circRNAs lack a 7-methylguanylate (m^7^G) cap.

During mRNA translation, the m^7^G cap recruits eukaryotic initiation factor 4E (eIF4E), which, in synergy with eIF4A and eIF4G, scaffolds the recruitment of other initiation factors and the ribosome^6^. In contrast, because circRNAs are covalently linked head to tail and lack a 5′ terminus, they must rely on cap-independent mechanisms, such as internal ribosome entry sites (IRESs), to initiate translation. Although the ability of circRNAs containing IRESs to encode proteins has long been known^7^, the principles of circRNA translation have yet to be thoroughly dissected. Identification of these principles is necessary to build better circRNA therapeutics and potentially surpass the translation capabilities of mRNA.

In this study, we created a modular high-throughput platform to make and test synthetic circRNAs. Using this platform, we systematically compare how circRNA expression is affected by factors including *N*^6^-methyladenosine (m^6^A) incorporation, vector topology, number of stop codons, 5′ and 3′ untranslated regions (UTRs), IRESs and synthetic aptamers. By optimizing and combining these elements for enhanced translation, we improve circRNA protein yields by several hundred-fold.

## Results

### Development of a modular circRNA assembly platform

**Fig. 1:**
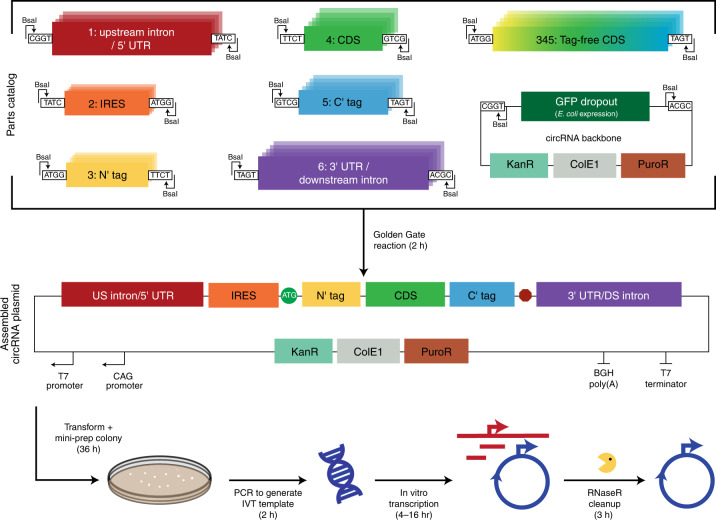
A modular cloning platform for circRNA enables rapid design–build–test cycles. Schematic describing the modular cloning platform used to create template plasmids for circRNA synthesis. Parts 1–6 corresponding to the upstream intron and 5′ UTR, IRES, N-terminal (N′) tag, coding sequence (CDS), C-terminal (C′) tag and 3′ UTR and the downstream intron were individually cloned into part plasmids via Golden Gate reactions (Supplementary Fig. 1). Part plasmids and the circRNA backbone were then combined in a second Golden Gate reaction to create a circRNA plasmid. The circRNA backbone contains a CAG promoter enabling circRNA transcription after transient transfection in cellulo, a T7 promoter enabling IVT, homology sequences that assist with RNA circularization, low-structure regions that facilitate RNaseR processivity and a bacterially expressed GFP dropout sequence to negatively select for incorrect assemblies. If a CDS without N′ or C′ tags was used, parts 3–5 were replaced with a single part. PCR products from circRNA plasmids were subsequently used as templates for IVT to synthesize RNA. Lastly, RNaseR cleanup was performed to digest linear RNAs and isolate circRNA. DS, downstream.

Synthesis of circRNAs via intron-assisted splicing and RNaseR digestion has been previously described^4^, but rapid creation of different circRNA species was difficult. To enable higher-throughput testing of circRNAs, we created a modular cloning platform consisting of a set of backbones and parts in a clearly defined and adaptable format compatible with both Golden Gate^8^ and Gibson cloning^9^ (Fig. 1 and Supplementary Fig. 1a). After various iterations of backbones, we arrived at a version incorporating a T7 promoter for in vitro transcription (IVT), the T4 thymidylate synthase (td) intron for RNA circularization, homology sequences to assist with circularization and low-structure regions to facilitate RNaseR digestion of precursor linear RNA. To assess circRNA translation across many conditions, we adopted a NanoLuc^10^ luminescence assay because of its broad quantitative range (Supplementary Fig. 1b), compatibility with a multi-well plate format and ability to measure both secreted and intracellular forms of NanoLuc. Using this platform, we systematically determined how aspects of circRNA design affect circRNA translation.

### m^6^A incorporation does not adversely affect circRNA translation

We previously showed that circRNAs can trigger immune responses in vivo that can be avoided by modifying circRNAs with m^6^A^4,11^. However, the effect of m^6^A incorporation on circRNA translation is unknown. To address this, we used our cloning platform to synthesize unmodified circRNAs encoding either NanoLuc or the fluorescent protein mNeonGreen. In separate preparations, we synthesized the same circRNAs with 5% m^6^A incorporation. Compared to unmodified circRNAs, circRNAs containing 5% m^6^A showed equivalent translation after transfection or electroporation in vitro (Supplementary Fig. 2a,b).

To gauge how m^6^A affected circRNA stability, we also performed an FBS degradation assay making use of the endogenous RNases in FBS (Supplementary Fig. 2c). CleanCap and 100% N^1^-methylpseudouridine (N^1^Ψ)-modified mRNA, the industry standard for mRNA-based therapies, was fully degraded by 1% FBS alongside unmodified circRNA. Conversely, circRNA containing 5% m^6^A was more resistant to nucleases and was not fully degraded until 2% FBS. These results indicate that 5% m^6^A incorporation does not adversely affect circRNA translation and may confer improved stability.

Given their reduced immunogenicity^11^, we focused our subsequent optimization efforts on m^6^A-modified circRNAs. Moving forward, we incorporated 5% m^6^A in every circRNA preparation unless otherwise stated.

### Vector topology and spacer requirements for circRNA translation

**Fig. 2:**
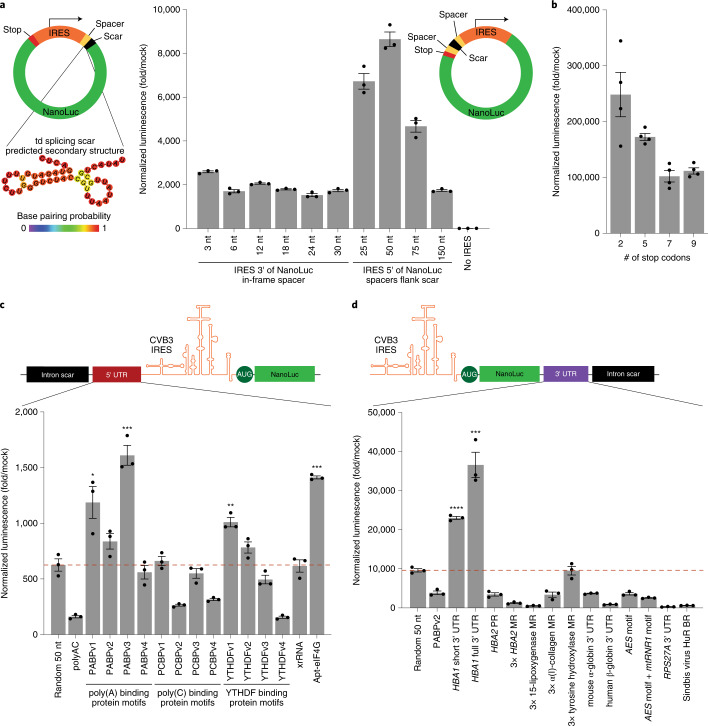
Optimization of RNA non-coding elements enable stronger circRNA translation. **a**, NanoLuc activity after transfection of HeLa cells with circRNAs containing either a 3′ or a 5′ IRES and spacer sequences of varying lengths. When the IRES is 3′ to the NanoLuc reporter, translation through the td splicing scar is unavoidable. The predicted secondary structure of this scar is shown. NanoLuc activity was normalized to constitutive firefly luciferase activity from the same sample and then divided by values from mock transfection. Data are mean ± s.e.m. for *n* = 3 biological replicates. **b**, NanoLuc activity at 24 hours after transfection of HeLa cells with circRNAs containing the indicated number of stop codons. NanoLuc activity was normalized to constitutive firefly luciferase activity from the same sample and then divided by values from mock transfection. Data are mean ± s.e.m. for *n* = 4 biological replicates. **c**, NanoLuc activity after transfection of HeLa cells with circRNAs containing different 5′ spacer sequences. NanoLuc activity was normalized to constitutive firefly luciferase activity from the same sample and then divided by values from mock transfection. Data are mean ± s.e.m. for *n* = 3 biological replicates. *P = 0.0213, **P = 0.0051 and ***P < 0.001 by unpaired two-sided *t*-test compared to a random 50-nt spacer sequence. **d**, NanoLuc activity after transfection of HeLa cells with circRNAs containing different 3′ UTR sequences. NanoLuc activity was normalized to constitutive firefly luciferase activity from the same sample and then divided by values from mock transfection. Data are mean ± s.e.m. for *n* = 3 biological replicates. ***P = 0.0012 and ****P < 0.0001 by unpaired two-sided *t*-test compared to a random 50-nt spacer sequence. BR, binding region; MR, minimal region; PR, protected region. Source data

We first sought to uncover principles behind circRNA vector topology that are necessary for strong translation. We began by synthesizing circRNAs with a coxsackievirus B3 (CVB3) IRES (denoted iCVB3) downstream, or 3′, of the reporter NanoLuc gene, maintaining the reading frame through the residual scar formed by the self-splicing reaction of the T4 td intron (Fig. 2a). In this orientation, translation through the splicing scar is unavoidable. Hypothesizing that the highly structured scar sequence might obfuscate the translation start site, we generated circRNA variants with in-frame spacers of varying lengths between the translation start and the splicing scar. The peptides encoded by these spacers reflected consensus viral leader peptide sequences from the rhinovirus family. Testing the expression of these circRNAs suggested that increasing the spacer length was non-beneficial for translation and that the ribosome was unaffected by the td splicing scar’s secondary structure.

We then reversed the topology of the circRNA vector, placing the IRES immediately upstream of the NanoLuc gene. Flanking this translation cassette, we tested adding spacers derived from random 50% GC content sequences of varying lengths in the 5′ and 3′ UTRs of the circRNA. When assayed for NanoLuc expression, we found that circRNAs with spacers 50 nucleotides (nt) in length yielded the strongest translation (Fig. 2a). We also tested whether the number of stop codons after the coding sequence affected circRNA expression, and we found that adding more than two stop codons (the number used in our cloning platform) reduced translation strength without affecting the size of the encoded protein (Fig. 2b and Supplementary Fig. 3a,b). Our results indicate that IRES-mediated translation of circRNAs can occur readily through an intron splicing scar, although with reduced efficiency compared to the IRES being directly upstream of a gene. Furthermore, translation of circRNAs can be improved by the addition of 50-nt spacers separating the IRES and gene of interest from the splicing scar.

### 5′ and 3′ UTRs can improve circRNA translation

5′ and 3′ UTRs in mRNAs can recruit RNA-binding proteins (RBPs) that enable strong translation as well as aspects of post-transcriptional regulation^6^. One such family of RBPs is poly(A)-binding proteins (PABPs), which interact with polyadenosine tracts of 12 nt or longer in the 3′ UTR and subsequently trigger binding of eIFs^12^. Other well-characterized RBPs include poly(C)-binding proteins (PCBPs), which recruit ribosomal proteins and *trans*-activating factors to picornavirus RNAs^13–17^, as well as YTHDF family members, which bind m^6^A and have been shown to regulate mRNA translation and stability^18,19^.

Previously, strong circRNA translation was reported using 5′ and 3′ UTR sequences consisting of ~50-nt sequences of mostly adenosine with interspersed cytosine—termed polyAC spacers^5^. We sought to understand if, instead of the random 50% GC content spacers that we used in our initial optimization, specific sequences could be installed to improve translation. We began our systematic dissection with the 5′ UTR region, which, in our case, refers to the sequence 5′ of the IRES, and synthesized 50-nt spacers encoding RNA-binding motifs for the three aforementioned RBP families, designing several versions per motif to account for sequence-specific variability. Additionally, we tested two highly structured sequences with well-defined effects: xrRNA, an RNA hairpin found in dianthoviruses that blocks degradation by the 5′-to-3′ exonuclease Xrn1 (ref. ^20^), and Apt-eIF4G, an eIF4G-recruiting aptamer that has been shown to increase mRNA translation when added to the 5′ UTR of transcripts^21^. Upon incorporating these sequences into the 5′ UTR of circRNAs and assaying for NanoLuc expression, we found that PABP motifs and the eIF4G-recruiting aptamer improved translation the most (Fig. 2c).

We then turned to optimizing the 3′ spacer downstream of the stop codons, drawing upon a wide array of 3′ UTRs with literature support for improving mRNA translation. These included the human α-globin 1 (*HBA1*) 3′ UTR in its shortened^22^ or full-length form^23^; the region of human α-globin 2 (*HBA2*) protected from RNase digestion by the α-complex, an RNA–protein complex implicated in mRNA stabilization^24^; minimal regions for α-complex binding to *HBA2*, rabbit 15-lipoxygenase, human α(I)-collagen or rat tyrosine hydroxylase tiled in triplicate^24^; the mouse α-globin 3′ UTR^25^; the human β-globin 3′ UTR truncated after the AAUAAA polyadenylation signal^26^; a motif from human amino-terminal enhancer of split (*AES*) alone or in combination with a motif from mitochondrially encoded 12S rRNA (mtRNR1)^27^; the 3′ UTR of mouse ribosomal protein S27a (*RPS27A*), which was highly expressed in Hep3B and 293T cells^28^; and the HuR-binding region from Sindbis virus that protects its transcript from RNase digestion^29^. When incorporated into circRNAs and assayed by NanoLuc expression, most of these 3′ UTRs that drive strong translation in an mRNA context failed to do so for circRNAs. However, replacing the 3′ spacer with either the short or full-length form of the *HBA1* 3′ UTR significantly improved translation strength (Fig. 2d).

### A full-length viral IRES is critical for strong translation

Viral IRESs are diverse and highly structured RNA regions found primarily in viral 5′ UTRs that promote cap-independent translation^30–32^. Because iCVB3, the baseline IRES used in our study, is nearly 750 nt, we sought to determine if it was possible to truncate an IRES while retaining circRNA translation. Structurally, iCVB3 can be divided into seven domains^33^, beginning with domain I containing a cloverleaf structure thought to be critical for viral replication^34^. Domains II–V have also been reported to interact with multiple IRES *trans*-activating factors (ITAFs)^35–37^, whereas domain VI hosts an AUG upstream of the true translation initiation site that recruits the 43S ribosomal pre-initiation complex^37–39^.

**Fig. 3:**
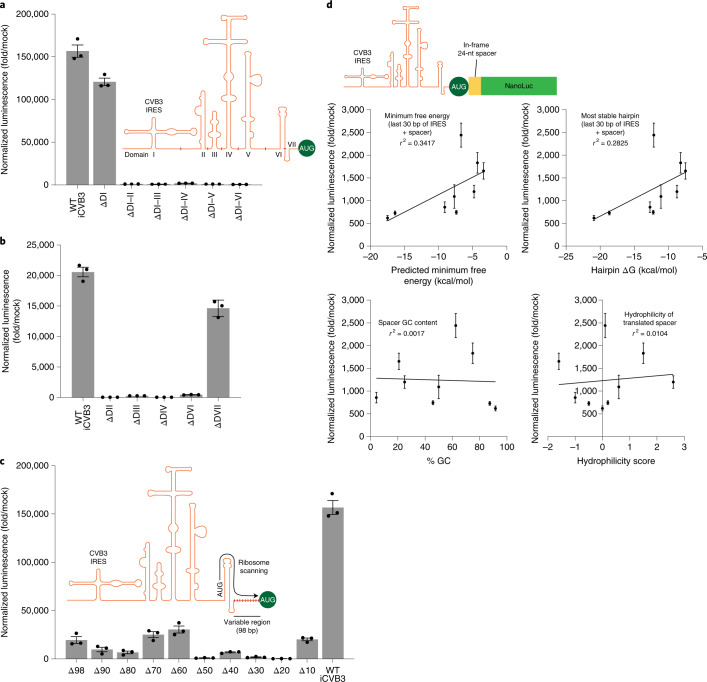
IRES truncations and the secondary structure of the IRES-coding sequence junction affect circRNA translation. **a**, NanoLuc activity at 24 hours after transfection of HeLa cells with circRNAs containing deletions of successive IRES domains starting from the 5′ end. Secondary structure and truncation points are indicated on the diagram. NanoLuc activity was normalized to constitutive firefly luciferase activity from the same sample and then divided by values from mock transfection. Data are mean ± s.e.m. for *n* = 3 biological replicates. **b**, NanoLuc activity at 24 hours after transfection of HeLa cells with circRNAs containing deletions of individual IRES domains. NanoLuc activity was normalized to constitutive firefly luciferase activity from the same sample and then divided by values from mock transfection. Data are mean ± s.e.m. for *n* = 3 biological replicates. **c**, NanoLuc activity at 24 hours after transfection of HeLa cells with circRNAs containing successive 10-nt deletions starting from the 3′ end of the IRES, immediately before the AUG start codon. NanoLuc activity was normalized to constitutive firefly luciferase activity from the same sample and then divided by values from mock transfection. Data are mean ± s.e.m. for *n* = 3 biological replicates. **d**, Correlations between the indicated properties and NanoLuc activity at 24 hours after transfection of HeLa cells with circRNAs containing different N-terminal leader sequences between the AUG start codon and NanoLuc reporter. NanoLuc activity was normalized to constitutive firefly luciferase activity from the same sample and then divided by values from mock transfection. Data are mean ± s.e.m. for *n* = 3 biological replicates. D, domain; WT, wild-type. Source data

We first performed IRES domain truncations starting from the 5′ end of iCVB3, choosing our truncations at boundaries where there was little known secondary structure base pairing. Compared to the full-length IRES, deletion of domain I cut circRNA translation by 23%, and further deletions eliminated translational activity (Fig. 3a). Deletions of other individual iCVB3 domains similarly reduced circRNA translation; removal of domain VII decreased luminescence by 29%, and loss of domain II, III, IV or VI completely ablated protein production (Fig. 3b). Finally, we performed successive truncations of iCVB3 from its 3′ end, a region highly variable in both sequence and length among different picornavirus IRESs that we hypothesized might be amenable to shortening. Unfortunately, 3′ deletion of as few as ten terminal nucleotides from this region severely reduced NanoLuc activity (Fig. 3c). Together, these data show that a full-length IRES is necessary for strong circRNA translation.

### IRES-coding sequence junction secondary structure affects translation strength

We next looked to understand coding sequence-specific factors that influence translation initiation in circRNAs. To assess this, we synthesized circRNAs with nine different 24-nt N-terminal leader sequences in frame between the AUG start codon and the NanoLuc reporter (Fig. 3d). We compared various features of these leader sequences—secondary structure, GC content and translated hydrophilicity—against the resulting NanoLuc reporter strength^40^. Indicators of secondary structure stability, such as predicted minimum free energy and free energy change for the most stable hairpin, were most correlated with NanoLuc translation, with 34.2% and 28.3% of translation strength variation explained by these factors, respectively. On the other hand, the GC content of the N-terminal leader and hydrophilicity of its encoded peptide were not predictive of translation efficiency. These findings suggest that in silico reduction of base-pairing interactions between the 3′ end of an IRES and 5′ end of a coding sequence can yield additional benefits for circRNA translation.

### Disruption of eIF4G binding to iCVB3 abrogates translation

**Fig. 4:**
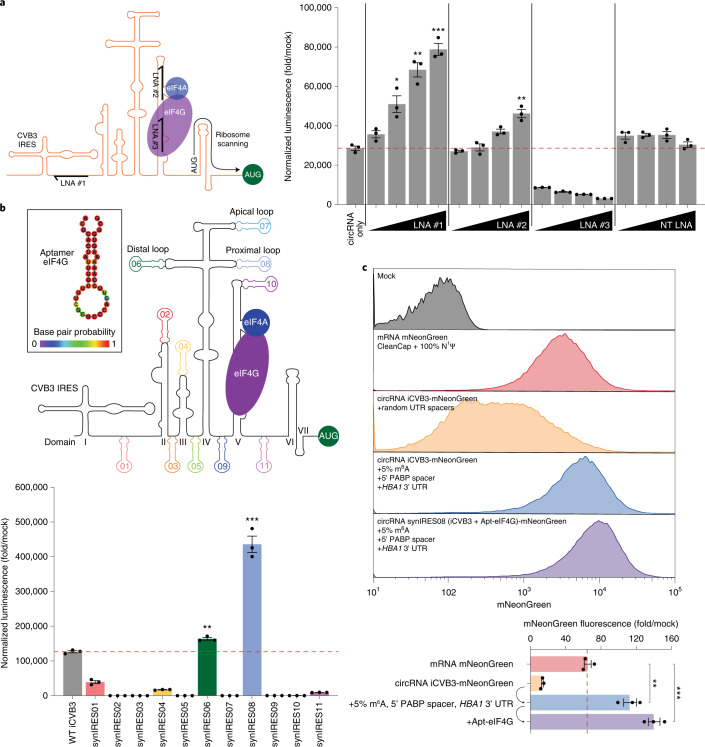
A synthetic IRES containing an eIF4G-recruiting aptamer drives stronger circRNA translation. **a**, NanoLuc activity at 24 hours after co-transfection of HeLa cells with circRNA and escalating doses (4.2–33.3 nM) of LNAs #1–3 or an NT LNA. LNAs #1–3 were designed to be complementary to regions of iCVB3 as indicated in the schematic. NanoLuc activity was normalized to constitutive firefly luciferase activity from the same sample and then divided by values from mock transfection. Data are mean ± s.e.m. for *n* = 3 biological replicates. *P = 0.0233, **P < 0.01 and ***P = 0.0001 by unpaired two-sided *t*-test compared to an equal dose of NT LNA. **b**, NanoLuc activity at 24 hours after transfection of HeLa cells with circRNAs containing an eIF4G-recruiting aptamer (Apt-eIF4G), shown in inset. Apt-eIF4G was inserted into iCVB3 at 11 different positions as indicated in the schematic. NanoLuc activity was normalized to constitutive firefly luciferase activity from the same sample and then divided by values from mock transfection. Data are mean ± s.e.m. for *n* = 3 biological replicates. **P = 0.0017 and ***P = 0.0002 by unpaired two-sided *t*-test compared to wild-type iCVB3. **c**, mNeonGreen fluorescence at 24 hours after electroporation of HEK293T cells with mRNA or circRNAs containing successive optimizations. mRNA was synthesized with CleanCap reagent, 100% N^1^Ψ incorporation and a 120-nt poly(A) tail. Mean mNeonGreen fluorescence was measured by flow cytometry and divided by values from mock electroporation. Data are histograms for *n* > 50,000 live singlet cells per condition and mean ± s.e.m. for *n* = 3 biological replicates. **P = 0.0044 and ***P = 0.0006 by unpaired two-sided *t*-test. For gating strategy, see Supplementary Fig. 10a. WT, wild-type. Source data

eIF4G and eIF4A binding to domain V of iCVB3 is thought to be a key step in initiating translation from this IRES^35^. Although it is unknown how these same eIFs contribute in the context of circRNAs, we hypothesized that interfering with their binding to iCVB3 might adversely affect translation. To block eIF-binding sites, we used locked nucleic acids (LNAs), which are modified nucleic acids with especially high antisense binding affinity that have previously been shown to disrupt IRES activity^41–43^. Specifically, we designed LNAs against a non-base-paired linker region between iCVB3 domains I and II (LNA #1), the footprint of eIF4A (LNA #2), the footprint of eIF4G (LNA #3) and a random non-targeting (NT) sequence (NT LNA) (Fig. 4a).

We tested the effect of LNAs across a range of concentrations, using NanoLuc as a readout for circRNA translation. As anticipated, NT LNA had minimal effect on the strength of iCVB3. In contrast, LNA #3 dose-dependently disrupted NanoLuc activity, implicating eIF4G sites in iCVB3 domain V as necessary for translating circRNAs. Unexpectedly, we also found that locking the secondary structure of the domain I–II junction with LNA #1 improved translation in a dose-dependent manner. Because RNA flexibility is a hallmark of picornavirus IRESs^32^, we theorize that this increase in translation strength may be due to fewer unfavorable base-pairing interactions between this region and the circRNA backbone. Interestingly, we observed a modest dose-dependent improvement rather than reduction in translation with LNA #2, suggesting that direct binding of eIF4A to iCVB3 domain V is not needed for circRNA translation. However, it is still possible that eIF4A in this context may directly interact with eIF4G.

We lastly synthesized four variants of iCVB3 with subdomain deletions of where eIF4G interacts with the upper stem of domain V (Supplementary Fig. 4b). These variants differed in the position where the stem loop was truncated, but, at a minimum, all ablated the eIF4G footprint. As expected, deletion of this key portion of iCVB3 domain V completely abrogated translational activity.

### Synthetic IRES engineering with an eIF4G-binding aptamer

From our LNA experiments, we concluded that eIF4G plays a pivotal role in initiating translation from IRESs in circRNAs. We, thus, hypothesized that engineering iCVB3 to have greater affinity for eIF4G might result in stronger circRNA translation. Apt-eIF4G, an eIF4G-recruiting aptamer, can improve cap-dependent translation when inserted in the 5′ UTR of mRNAs^21^. We designed synthetic variants of the iCVB3 where we rationally inserted Apt-eIF4G at hypothetically permissible regions within the IRES (Fig. 4b). These positions were either within the flexible non-base-paired inter-domain regions (synIRES01, 03, 05, 09 and 11), which were chosen to avoid aberrant Apt-eIF4G-linker interactions, or at the end of loop domains (synIRES02, 04, 06, 07, 08 and 10), with removal of several wild-type nucleotides to smoothly transition from the stem–loop structure into Apt-eIF4G’s RNA stem. In all cases, rational engineering choices were informed by in silico RNA structure prediction (Supplementary Fig. 5)^40^. Using our NanoLuc assay, we found that domain IV’s cruciform structure was the most permissive to Apt-eIF4G insertion. Both synIRES06 and synIRES08, where Apt-eIF4G was inserted in the distal and proximal loops of domain IV, respectively, showed significantly improved translation over wild-type iCVB3. Conversely, insertion at the apical loop of domain IV completely abrogated translation, consistent with reports of an essential internal C-rich loop and GNRA tetraloops at this site^44,45^.

We tested the generalizability of our results by switching the reporter to mNeonGreen, a monomeric green fluorescent protein (GFP). Compared to CleanCap and 100% N^1^Ψ-modified mRNA or unmodified circRNA with random 5′ and 3′ UTRs, 5% m^6^A-modified circRNA with the 5′ PABP spacer and *HBA1* 3′ UTR exhibited greater mNeonGreen expression (Fig. 4c). This was further improved by aptamer engineering of iCVB3 to include Apt-eIF4G.

We additionally attempted to rescue iCVB3 domain V eIF4G footprint deletions through insertion of Apt-eIF4G in the proximal loop of domain IV (Supplementary Fig. 4b). However, no recovery of translation was achieved by this strategy for any of the four variants. Prior toe-printing analysis deduced conformational changes in domain VI and the 3′ end of iCVB3 following the recruitment of eIF4G and eIF4A^35^. Our results suggest that these RNA conformational changes are indeed crucial for proper ribosome assembly and that simply recruiting eIF4G locally is insufficient for translation initiation.

### Identification of robust higher-strength IRESs

**Fig. 5:**
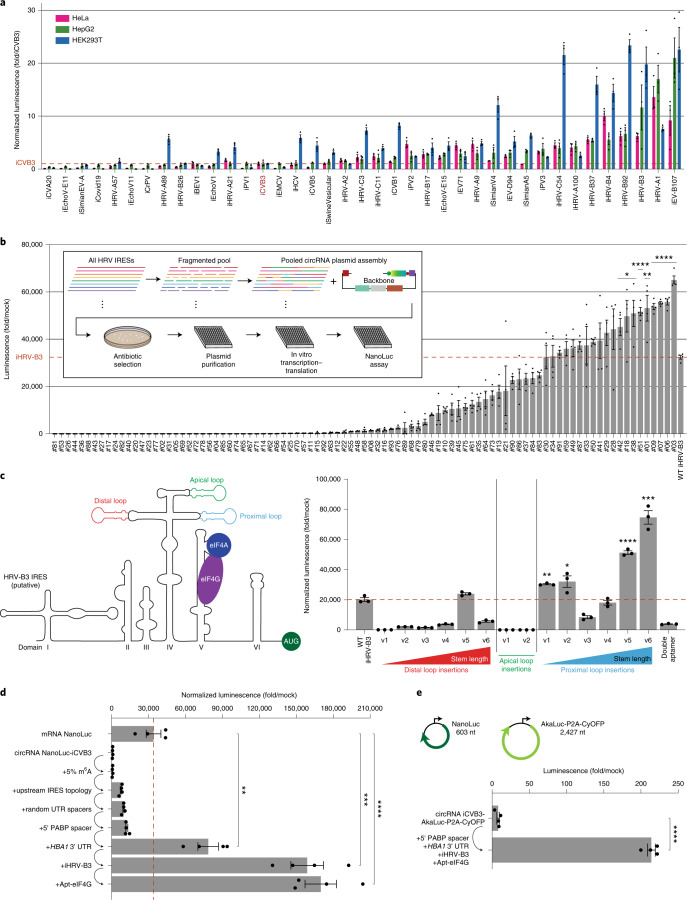
Large-scale screens and IRES engineering expand the repertoire of strong IRESs. **a**, NanoLuc activity at 24 hours after transfection of HeLa, HepG2 and HEK293T cells with circRNAs containing the indicated IRESs. NanoLuc activity was normalized to constitutive firefly luciferase activity from the same sample and then divided by values from mock transfection. Data are mean ± s.e.m. for *n* = 3 biological replicates. **b**, NanoLuc activity after IVTT of circRNA plasmids containing shuffled human rhinovirus IRESs. NanoLuc activity was divided by values from mock IVTT. Data are mean ± s.e.m. for *n* = 4 biological replicates. *P < 0.05, **P = 0.0095 and ****P < 0.0001 by unpaired two-sided *t*-test compared to wild-type iHRV-B3. **c**, NanoLuc activity at 24 hours after transfection of HeLa cells with circRNAs containing different insertions of Apt-eIF4G into iHRV-B3. The putative iHRV-B3 secondary structure, predicted eIF4G and eIF4A binding sites and Apt-eIF4G insertion locations are shown. Versions (v1–v6) of each insertion differ in stem length. Double aptamer refers to Apt-eIF4G insertion at both distal and proximal loops. NanoLuc activity was normalized to constitutive firefly luciferase activity from the same sample and then divided by values from mock transfection. Data are mean ± s.e.m. for *n* = 3 biological replicates. *P = 0.0422, **P = 0.0018, ***P = 0.0003 and ****P < 0.0001 by unpaired two-sided *t*-test compared to wild-type iHRV-B3. **d**, NanoLuc activity at 24 hours after transfection of HeLa cells with mRNA or circRNAs containing successive optimizations. mRNA was synthesized with CleanCap reagent, 100% N^1^Ψ incorporation and a 120-nt poly(A) tail. NanoLuc activity was normalized to constitutive firefly luciferase activity from the same sample and then divided by values from mock transfection. Data are mean ± s.e.m. for *n* = 4 biological replicates. **P = 0.0051, ***P = 0.0001 and ****P < 0.0001 by unpaired two-sided *t*-test. **e**, AkaLuc activity at 24 hours after electroporation of HeLa cells with circRNAs encoding AkaLuc-P2A-CyOFP. CircRNA iCVB3-AkaLuc-P2A-CyOFP was synthesized with 5% m^6^A, upstream IRES topology and random UTR spacers. AkaLuc activity was divided by values from mock electroporation. Sizes indicate coding sequence lengths for NanoLuc and AkaLuc-P2A-CyOFP. Data are mean ± s.e.m. for *n* = 4 biological replicates. ****P < 0.0001 by unpaired two-sided *t*-test. WT, wild-type. Source data

IRESs have evolved a variety of mechanisms to utilize host factors for initiating translation. To further optimize circRNA expression, we sought to find IRESs with stronger translation than those previously described in the literature^5,46^. Over several rounds of synthesis and testing, we characterized a number of IRESs spanning different types and species in circRNAs. We began with IRESs representing canonical IRES types (type in parenthesis), such as from CVB3 (1), poliovirus 1 (PV1) (1), human rhinovirus A1 (HRV-A1) (1), encephalomyocarditis virus (EMCV) (2), hepatitis C virus (HCV) (3) and cricket paralysis virus (CrPV) (4). We noticed that type 1 IRESs appeared to drive strong translation in the context of circRNAs (Fig. 5a), matching expectations as these IRESs have extended structures that may allow them to scaffold a full set of ITAFs to initiate translation^31^. We, thus, expanded our screen to include a large set of putative type 1 IRESs from the enterovirus genus, which we incorporated into circRNAs and assayed for NanoLuc translation.

In our screen, we identified many IRESs with stronger translation than iCVB3 across multiple cell lines (Fig. 5a). In particular, IRESs from the human rhinovirus B (HRV-B) and enterovirus B (EV-B) species, such as iHRV-B3 and iEV-B107, drove robust circRNA translation. To validate this result with a different transgene, we used a fluorescent reporter assay to assess Cre-mediated recombination after transfection of circRNAs encoding Cre recombinase (Supplementary Fig. 6). At 24 hours after transfection, we observed greater recombination with iHRV-B3 compared to iCVB3, supporting iHRV-B3 as a stronger IRES for circRNA translation.

With this knowledge, we synthesized IRESs from every HRV-B and EV-B subspecies with a publicly available sequence on NCBI Virus (http://ncbi.nlm.nih.gov/labs/virus) and incorporated them into circRNA expression plasmids. Given the scale of this screen, we opted for an in vitro coupled transcription–translation (IVTT) approach, using circRNA expression plasmids rather than purified circRNAs as the input material (Supplementary Fig. 7a). In the IVTT-based NanoLuc assay, we found a large number of HRV-B and EV-B IRESs with greater translational activity than iCVB3. We validated some of these IRESs in cellulo using purified circRNAs (Supplementary Fig. 7b). Although many hits turned out to be false positives, our discovery of iHRV-B92 and iHRV-B97 as higher-strength IRESs was recapitulated. When these same IRESs were also tested in a linear RNA format, relative differences in translation strength held but with a 100-fold reduction in absolute expression compared to circRNAs (Supplementary Fig. 7b). For the strongest IRESs, we tested NanoLuc translation in four different cell lines and found that many drove efficient translation independent of cell type (Supplementary Fig. 7c). At the same time, some IRESs demonstrated stronger translation in a specific cell type, such as iHCV and iHRV-C54 in HEK293T cells and iHRV-A100 and iHRV-B4 in KG-1 cells.

### Synthetic IRES engineering through unbiased DNA shuffling

DNA shuffling is an unbiased approach commonly used to generate large diverse libraries for selecting novel engineered proteins^47^. Shuffling particularly makes sense over other library-generating strategies, such as point mutagenesis, when a homologous family of related proteins is available to act as seed templates for the shuffling reaction. Because we observed the strongest translation overall with IRESs from HRV, we performed DNA shuffling by fragmenting 41 HRV IRESs and cloning the resulting pool into circRNA plasmids. (Fig. 5b). We isolated 93 circRNA expression plasmids with unique shuffled IRESs and measured their translation strength using an IVTT assay, with iHRV-B3 as an internal benchmarking control. From these 93 shuffled IRESs, we identified nine with significantly stronger translational activity than wild-type iHRV-B3, illustrating the ability of IRES shuffling to engineer improved IRESs for circRNA applications.

### Validation of Apt-eIF4G IRES engineering with iHRV-B3

We hypothesized that our aptamer-engineering approach with Apt-eIF4G might also improve translation for IRESs of indeterminate structure. To test this, we took a strong IRES, iHRV-B3, and attempted to predict its domain architecture in silico^40^, which identified six domains, including a cruciform structure in domain IV (Fig. 5c). We focused on loops within this cruciform structure and performed Apt-eIF4G insertions at the distal, apical and proximal loop locations, varying the length of the resulting stem by rationally inserting base-paired RNA nucleotides and validating the structure in silico. We reasoned that, by assessing a range of stem lengths, we might uncover a particular position for Apt-eIF4G most favorable to cooperative binding effects. Indeed, we found that Apt-eIF4G insertions at the proximal loop of domain IV significantly improved circRNA translation compared to wild-type iHRV-B3, demonstrating the broader utility of our aptamer-engineering strategy to synthesize stronger IRESs. As with iCVB3, apical loop insertions of Apt-eIF4G also destroyed iHRV-B3 activity, consistent with a predicted GNRA tetraloop in this region. Although we attempted to perform a double-aptamer insertion of Apt-eIF4G at both the distal and proximal loops, this greatly reduced circRNA translation.

### Quantification of combined circRNA optimizations

We examined each of our earlier circRNA optimizations and compared them in a single experiment (Fig. 5d). We began with iCVB3 downstream of NanoLuc and successively incorporated m^6^A, reversed the vector topology, added random 5′ and 3′ UTR spacers, modified the 5′ spacer to include a PABP motif, replaced the 3′ UTR spacer with the *HBA1* 3′ UTR, switched the IRES to iHRV-B3 and inserted a proximal loop aptamer into iHRV-B3. We found that these changes progressively increased circRNA expression without compromising RNA yield or circularization efficiency (Supplementary Fig. 8a,b), with the final design exhibiting a 224-fold improvement relative to unoptimized circRNA and significantly more translation than CleanCap and 100% N^1^Ψ-modified mRNA.

To validate our findings with a larger transgene, we then synthesized circRNAs expressing AkaLuc-P2A-CyOFP, a coding sequence more than four times longer than NanoLuc (Fig. 5e). When assayed for Aka luciferase (AkaLuc) activity, the combined additions of a 5′ PABP spacer, *HBA1* 3′ UTR, HRV-B3 IRES and proximal loop Apt-eIF4G insertion again improved circRNA translation, supporting the generalizability of these optimizations.

Finally, to evaluate the kinetics of circRNA translation, we compared secreted NanoLuc levels from cells electroporated with either CleanCap and 100% N^1^Ψ-modified mRNA or 5% m^6^A-modified circRNA driven by iHRV-B3 (Supplementary Fig. 9). The secretion tag incorporated in the NanoLuc reporter allowed us to repeatedly harvest media to measure translation over a time course. We found that circRNA and mRNA translation kinetics differed substantially, with circRNA taking over 24 hours to reach its maximum translation strength. Consistent with previous data on the long-lived nature of circRNAs^48^, we also saw that the duration of circRNA translation greatly exceeded that of mRNA.

### In vivo expression of optimized circRNAs

**Fig. 6:**
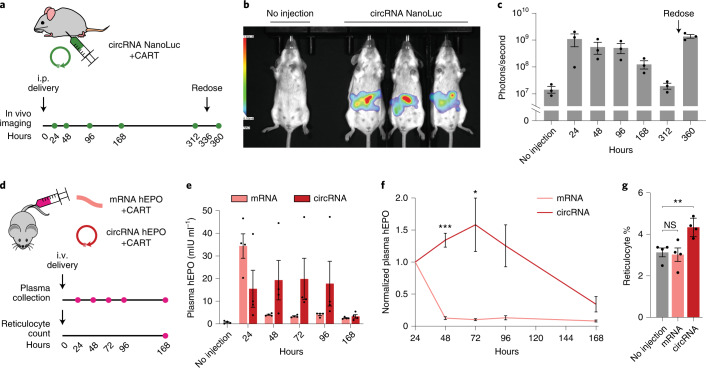
Engineered circRNAs demonstrate more durable translation and functional activity in vivo. **a**, CircRNA with 5% m^6^A incorporation encoding NanoLuc was synthesized with the following optimizations: upstream IRES topology, 5′ PABP spacer, *HBA1* 3′ UTR and HRV-B3 IRES with proximal loop Apt-eIF4G insertion. CircRNAs were formulated for intraperitoneal delivery in mice using CARTs. Expression was assayed using an optical imaging system after intraperitoneal injections of the fluorofurimazine substrate at the indicated timepoints. At 336 hours (14 days) after circRNA NanoLuc administration, mice were redosed. **b**, In vivo luminescence image of an untreated mouse (left) versus mice receiving circRNA NanoLuc (right) at 24 hours after dosing. **c**, Quantification of luminescence per mouse at different timepoints after circRNA NanoLuc administration. Redosing was performed at 336 hours (14 days). Data are mean ± s.e.m. for *n* = 3 animals per condition. **d**, CircRNA with 5% m^6^A incorporation encoding hEPO was synthesized with the following optimizations: upstream IRES topology, 5′ PABP spacer, *HBA1* 3′ UTR and HRV-B3 IRES with proximal loop Apt-eIF4G insertion. mRNA-encoding hEPO was synthesized with CleanCap reagent, 100% N^1^Ψ incorporation and a 120-nt poly(A) tail. Equimolar doses of circRNA and mRNA were formulated for intravenous delivery in mice using CARTs. Plasma hEPO was measured by ELISA in one cohort at the indicated timepoints. Reticulocytes were counted in a separate cohort at 168 hours (7 days). **e**, Quantification of plasma hEPO at different timepoints after circRNA hEPO or mRNA hEPO administration. Data are mean ± s.e.m. for *n* = 4 animals per condition. **f**, Plasma hEPO expression normalized to the 24-hour level of each mouse at different timepoints after circRNA hEPO or mRNA hEPO administration. Data are mean ± s.e.m. for *n* = 4 animals per condition. *P = 0.0487 and ***P = 0.0001 by unpaired two-sided *t*-test with Bonferroni correction compared to mRNA. **g**, Reticulocyte percentage among red blood cells at 168 hours after circRNA hEPO or mRNA hEPO administration. Data are mean ± s.e.m. for *n* = 4 animals per condition. **P = 0.0080 by unpaired two-sided *t*-test. NS, not significant. For gating strategy, see Supplementary Fig. 10b. i.p., intraperitoneal; i.v., intravenous. Source data

We combined the above circRNA optimizations—upstream IRES topology, 5′ PABP spacer, *HBA1* 3′ UTR and HRV-B3 IRES with proximal loop Apt-eIF4G insertion—together to test the expression of circRNAs in vivo. To deliver RNAs, we formulated them with charge-altering releasable transporters (CARTs), which are temporarily cationic molecules capable of mediating mRNA expression in mice^49,50^. We first administered circRNAs encoding NanoLuc in mice via intraperitoneal injections (Fig. 6a,b). Compared to untreated animals, those receiving circRNA showed greater luminescent activity for at least 1 week (Fig. 6c), indicating that engineered circRNAs can be expressed in vivo. When redosed 2 weeks after the first injection, NanoLuc expression was also indistinguishable from initial levels (Fig. 6c), suggesting that repeat administration of circRNAs may be feasible.

We then performed a head-to-head comparison of optimized circRNA versus CleanCap and 100% N^1^Ψ-modified mRNA in vivo using RNAs encoding human erythropoietin (hEPO), a secreted protein used to treat anemia. After intravenous administration in mice via CARTs, plasma hEPO levels from circRNA were initially less than those from mRNA (Fig. 6d,e). However, compared to mRNA expression of hEPO, which rapidly declined within 48 hours, circRNA expression remained consistent until at least 96 hours after injection (Fig. 6e,f). Functionally, hEPO can elevate reticulocyte production in mice, although much higher concentrations are required than for mouse EPO^51^. Reticulocyte counts were significantly increased in mice that received a single dose of hEPO-encoding circRNA after 1 week, whereas reticulocyte levels after an equimolar dose of mRNA were no different than those from untreated animals. Together, our data show that engineered circRNAs can express at strengths similar to modified mRNAs in vivo but with greater duration.

## Discussion

RNA circularization has the potential to transform RNA-based medicines by extending the durability of these otherwise highly transient molecules. However, because the fundamental mechanisms of circRNA and mRNA translation differ, decades of knowledge on how to maximize mRNA translation may not necessarily apply to circRNAs. Although protein expression from circRNAs has been demonstrated previously^5^, the syntax of circRNA translation is not fully characterized. In this study, we attempted to decode this syntax and devised multiple generalizable strategies for circRNA engineering that improve translation. To enable our study, we created a circRNA modular cloning platform that allowed for testing of numerous sequence variations and independent optimization of multiple parameters. Although we designed most of our sequences rationally, our platform can be used for random library generation, as we demonstrated with IRES shuffling. Independent libraries can also be modularly assembled to produce rich RNA element datasets, such as combining shuffled 5′ UTR and shuffled 3′ UTR regions to flank a reporter gene.

Using this platform, we identified several approaches to improve protein translation from circRNAs, some of which may prove useful for engineering RNAs more broadly. In particular, we found that LNA-based disruption of secondary structure can ablate or enhance translation initiation in circRNAs. Because IRES-driven translation is highly dependent on RNA structure, antisense oligos that interfere with structural elements can provide targeted control over an IRES. We showed that a LNA targeting the natural footprint of eIF4G eliminated IRES translation in a circRNA. On the other hand, a LNA locking the conformation of a flexible region boosted circRNA translation, possibly by limiting the formation of unfavorable secondary structures. Our data indicate that antisense oligos can offer an axis of control over IRES and circRNA functionality. For instance, if a circRNA is producing an undesirable protein, translation can be readily halted using LNAs.

We also demonstrated that recruiting eIF4G to an IRES using aptamers can readily enhance circRNA translation. Correct positioning of the aptamer required minimally disrupting native RNA structures while placing it in proximity to the translation initiation core of the IRES. Future optimizations could adapt RNA aptamers toward solving other needs, such as enabling small-molecule control over circRNA translation or directing circRNAs toward specific intracellular targets. Additionally, incorporation of RNA aptamers might provide an avenue for cell-type-specific expression of circRNAs.

We further observed that unbiased IRES shuffling can create libraries of novel IRESs with varying strengths. This approach can vastly expand the repertoire of usable IRESs and may allow for delivery of circRNAs with finely tuned translational activities that parallel physiological expression. Interestingly, translation from a given IRES can differ by 100-fold depending on whether the RNA is circular or linear. This is consistent with a recent screen for sequences driving cap-independent translation in circular and linear RNAs^42^ and suggests that there are mechanisms of translational control unique to circRNAs.

Combining these and other design principles, we found that engineered circRNAs can produce more protein than mRNAs in vitro and exhibit greater durability of translation both in vitro and in vivo. Moreover, redosing of circRNAs after 2 weeks showed no loss in expression compared to the initial dose, supporting the feasibility of administering circRNAs in the same subject multiple times. In humans, normal EPO levels range from 2.8 mIU ml^−1^ to 17.9 mIU ml^−1^ (ref. ^52^). Using circRNA delivered with CARTs, these levels were achieved for at least 4 days in mice and achieved a functional effect on reticulocyte production. As CARTs are designed to be used with mRNA and were not optimized for circRNA transport, further improvement of circRNA delivery methods may yield even greater translation.

In summary, we systematically dissected five functional elements controlling circRNA translation: vector topology, 5′ and 3′ UTRs, IRESs and synthetic aptamers. When optimized, these components increase circRNA protein yields by several hundred-fold and enable potent and durable protein production in vivo.

## Methods

### Molecular cloning

Part plasmids (Supplementary Fig. 1) were synthesized by cloning polymerase chain reaction (PCR) products or pre-made DNA fragments (Integrated DNA Technologies) into a custom entry vector (pRC0569**)** via a Golden Gate reaction. The BsmBI-v2 Golden Gate reaction was set up and performed following the manufacturer’s instructions (New England Biolabs (NEB), E1602L). Turbo Competent (NEB) cells were transformed using 2 µl of the reaction and plated onto carbenicillin agar plates. Non-green colonies were picked, mini-prepped and sequenced.

CircRNA plasmids were assembled by cloning parts 1–6 into a custom backbone (pRC0940) via a second Golden Gate reaction. The BsaI Golden Gate reaction was set up and performed following the manufacturer’s instructions (NEB, E1601L). Turbo Competent (NEB) cells were transformed using 2 µl of the reaction and plated onto kanamycin agar plates. Non-green colonies were picked, mini-prepped and sequenced.

Sequences for backbones pRC0569 and pRC0940 and all parts are listed in Supplementary Table 1.

### CircRNA synthesis

CircRNAs were synthesized by IVT using the HiScribe T7 High Yield RNA Synthesis Kit (NEB). IVT templates were PCR amplified for 30 cycles using forward and reverse circRNA oligos (Supplementary Table 1) and column purified before RNA synthesis. One microgram of circRNA template was used per 20 µl of IVT reaction. Reactions were incubated overnight at 37 °C with shaking at 1,000 r.p.m. with a heated lid. IVT templates were subsequently degraded with 2 µl of DNaseI per IVT reaction for 20 minutes at 37 °C with shaking at 1,000 r.p.m. The remaining RNA was column purified before further enzymatic reactions.

To isolate circRNAs, column-purified RNA was digested with one unit of RNaseR per microgram of RNA for 60 minutes at 37 °C with shaking at 1,000 r.p.m. Samples were then column purified, quantified using a NanoDrop One spectrophotometer and verified for complete digestion using an Agilent TapeStation. In some instances, due to reagent shortages, verification was performed with agarose gel under formamide-based denaturing conditions (NEB, B0363S). In cases of incomplete digestion of linear RNAs, RNaseR digestion was repeated.

### mRNA synthesis

IVT templates for mRNA synthesis were PCR amplified for 30 cycles using forward and reverse mRNA oligos (Supplementary Table 1) and column purified before RNA synthesis. mRNA was then synthesized using the HiScribe T7 High Yield RNA Synthesis Kit (NEB) with the following modifications: CleanCap AG (TriLink N-7113) was added to a final concentration of 4 mM, and N^1^Ψ (TriLink N-1019) was fully substituted for UTP.

One microgram of mRNA template was used per 20 µl of IVT reaction. Reactions were incubated for 2 hours at 37 °C with shaking at 1,000 r.p.m. with a heated lid. IVT templates were subsequently degraded with 2 µl of DNaseI per IVT reaction for 20 minutes at 37 °C with shaking at 1,000 r.p.m. The remaining mRNA was column purified before use.

### Cell culture and transfection

HeLa (CCL-2), HEK293T (CRL-11268), HepG2 (HB-8065) and KG-1 (CCL-246) cells from the American Type Culture Collection were maintained with DMEM (Thermo Fisher Scientific) supplemented with 10% FBS (Gibco) and 1% penicillin–streptomycin (Gibco). Cell lines were not authenticated. For routine subculture, 0.25% TrypLE (Thermo Fisher Scientific) was used for cell dissociation.

RNA delivery was achieved with TransIT-mRNA transfection, Lipofectamine transfection or NEON electroporation. Within each experiment, the molar amount of mRNA or circRNA delivered and transfection method used was the same for all samples unless otherwise indicated. For TransIT-mRNA transfections, 3 µl of TransIT-mRNA reagent (Mirus Bio) was used per microgram of RNA. Besides this change, RNA delivery was performed following the manufacturer’s instructions.

### In vitro NanoLuc assay

Cells were electroporated with the pGL4.54 [luc2/TK] vector (Promega) expressing firefly luciferase and transfected with mRNA or circRNA 48 hours later. At 24 hours after transfection, cells were harvested in 100 µl of passive lysis buffer (Promega) and lysed by rocking and pipetting for roughly 15 minutes at room temperature. Lysate was centrifuged at 4,000*g* for 10 minutes to clear debris, and 5 µl of clarified lysate was transferred into a 384-well white-bottom assay plate (PerkinElmer). To each well, 10 µl of ONE-Glo EX from the Promega Nano-Glo Dual-Luciferase Reporter Assay System was added, after which the plate was vortexed for 1 minute, incubated at room temperature for an additional 2 minutes and read on a TECAN M1000 Infinite Pro microplate reader using i-control 1.10 software with an integration time of 1,000 ms.

Samples were first measured for firefly luminescence, which was used as a constitutive control. To each well, 10 µl of freshly made NanoDLR Stop & Glo Reagent was then added, after which the plate was vortexed for 1 minute and incubated at room temperature for an additional 9 minutes before NanoLuc luminescence was read. Normalized luminescence per well was calculated by dividing the signal from NanoLuc by that from firefly luciferase. Within each experiment, normalized luminescence was displayed in terms of fold change relative to mock (no RNA) transfections.

### mNeonGreen flow cytometry assay

CircRNAs and mRNAs expressing mNeonGreen^53^ with different optimizations were electroporated into HEK293T cells via NEON electroporation. At 24 hours after electroporation, cells were lifted using warmed TrypLE (Thermo Fisher Scientific), which was quenched with DMEM (Thermo Fisher Scientific) and incubated in PBS containing DAPI (Thermo Fisher Scientific) for dead cell exclusion. Cells were acquired on an Attune NxT flow cytometer with the same voltages applied to all conditions and analyzed using FlowJo 10 software. At least 50,000 live singlet cells were recorded per sample.

### IVTT

Coupled IVTT was performed using the 1-Step Human Coupled IVT Kit (Thermo Fisher Scientific) following the manufacturer’s instructions. In brief, circRNA plasmids were incubated with HeLa lysate, accessory proteins and the reaction mix for at least 90 minutes. An aliquot from each reaction was then used to measure NanoLuc activity as described above.

### AkaLuc assay

CircRNAs expressing AkaLuc-P2A-CyOFP^54^ with different optimizations were electroporated into HeLa cells via NEON electroporation and plated in a 96-well plate. At 24 hours after electroporation, cells were washed with PBS and incubated with 100 µl of TokeOni AkaLumine-HCl substrate (Sigma-Aldrich) diluted to 250 µM in Opti-MEM (Gibco) for 5 minutes at room temperature. Luminescence was read on a SpectraMax M5 Microplate Reader (Molecular Devices) using SoftMax Pro 7.1 software with an integration time of 1,000 ms.

### CART synthesis

O_6_-stat-N_6_:A_9_ CARTs, consisting of a 1:1 mixture of oleyl (O) and nonenyl-substituted (N) carbonate monomers, followed by a block of α-amino ester monomers (A), were prepared as previously described^55^. End group analysis of the polymer confirmed block lengths of 6 nonenyl and 6 oleyl carbonate units and 9 cationic amino-ester units.

### In vivo delivery of circRNA and mRNA

All animal experiments were performed in 2–6-month-old female BALB/c mice obtained from The Jackson Laboratory. To formulate RNAs, 10.7 ng per nt of linear or circular RNA (equivalent to 10 µg of hEPO mRNA) was diluted in pH 5.5 PBS, mixed with O_6_-stat-N_6_:A_9_ CARTs at a 10:1 cation:anion ratio and immediately injected either intraperitoneally or intravenously via the tail vein. Particle sizes for CART/circRNA complexes were ~170 nm. A total volume of 150 µl was used per injection. All experimental procedures were approved by the Institutional Animal Care and Use Committee at Stanford University and performed in adherence to the National Institutes of Health Guide for the Care and Use of Laboratory Animals.

### NanoLuc in vivo imaging

In vivo NanoLuc activity was measured using an Ami HT optical imaging system (Spectral Instruments). At each timepoint, mice were anesthetized with isoflurane and intraperitoneally injected with 200 µl of the fluorofurimazine substrate (Promega) reconstituted in 2.1 ml of PBS per vial. Mice were imaged after 10 minutes using default settings and an exposure time of 10 seconds. Luminescent activity was quantified using Aura 4.0 imaging software.

### hEPO ELISA assay

hEPO levels in mice were measured using the SimpleStep Human Erythropoietin ELISA Kit (Abcam). At each timepoint, approximately 100 µl of blood was collected in heparinized capillary tubes from the tail vein of each mouse and transferred into an EDTA-coated tube. Blood was centrifuged at 2,000*g* for 10 minutes with the resulting plasma used as input for the ELISA. Final concentrations for hEPO were adjusted based on the volume of plasma measured.

### Reticulocyte counts

Reticulocytes in peripheral mouse blood were measured using the Reticulocyte Reagent System (BD Biosciences), which uses thiazole orange to label reticulocytes. In brief, 10 µl of blood was collected from the tail vein of each mouse and immediately mixed with 1 ml of the reagent. After incubating in the dark at room temperature for 30 minutes, samples were analyzed on a BD LSR II flow cytometer with 100,000 events recorded per sample. Reticulocytes were defined as singlet red blood cells positive for thiazole orange.

### Reporting summary

Further information on research design is available in the Nature Research Reporting Summary linked to this article.

## Online content

Any methods, additional references, Nature Research reporting summaries, source data, extended data, supplementary information, acknowledgements, peer review information; details of author contributions and competing interests; and statements of data and code availability are available at 10.1038/s41587-022-01393-0.

## Supplementary information

Supplementary Information Supplementary Figs. 1–10 and Supplementary Methods

Reporting Summary

Supplementary Table Sequence information

## Data Availability

Source data for figures are provided in the article. All reagents generated in this study are available upon reasonable request. Source data are provided with this paper.

## Source data

Source Data Fig. 2 Statistical Source Data
Source Data Fig. 3 Statistical Source Data
Source Data Fig. 4 Statistical Source Data
Source Data Fig. 5 Statistical Source Data
Source Data Fig. 6 Statistical Source Data
