# Author Correction: Engineering circular RNA for enhanced protein production

**DOI:** 10.1038/s41587-022-01472-2

**Published:** 2022-08-17

**Authors:** Robert Chen, Sean K. Wang, Julia A. Belk, Laura Amaya, Zhijian Li, Angel Cardenas, Brian T. Abe, Chun-Kan Chen, Paul A. Wender, Howard Y. Chang

**Affiliations:** 1 Center for Personal Dynamic Regulomes, Stanford University, Stanford, CA USA; 2 Department of Ophthalmology, Stanford University School of Medicine, Stanford, CA USA; 3 Department of Computer Science, Stanford University, Stanford, CA USA; 4 Department of Chemistry, Stanford University, Stanford, CA USA; 5 Department of Chemical and Systems Biology, Stanford University, Stanford, CA USA; 6 Howard Hughes Medical Institute, Stanford University, Stanford, CA USA

**Keywords:** RNA splicing, Gene therapy, Gene delivery, Nucleic-acid therapeutics

Correction to: *Nature Biotechnology* 10.1038/s41587-022-01393-0, published 18 July 2022.

In the version of this article initially published, several sequences in Supplementary Table 1 were listed incorrectly, which have now been replaced in the online version of the article.

