# Supplementary material for: Engineering circular RNA for enhanced protein production
Source: Nat Biotechnol. 2022 Jul 18;41(2):262–72. doi: 10.1038/s41587-022-01393-0 (PMC9931579; doi:10.1038/s41587-022-01393-0)
Supplement: Supplementary file 1 — Supplementary Figs. 1–10 and Supplementary Methods [file 41587_2022_1393_MOESM1_ESM.pdf]

nature portfolio

https://doi.org/10.1038/s41587-022-01393-0

Supplementary information

Engineering circular RNA for enhanced protein production

In the format provided by the authors and unedited

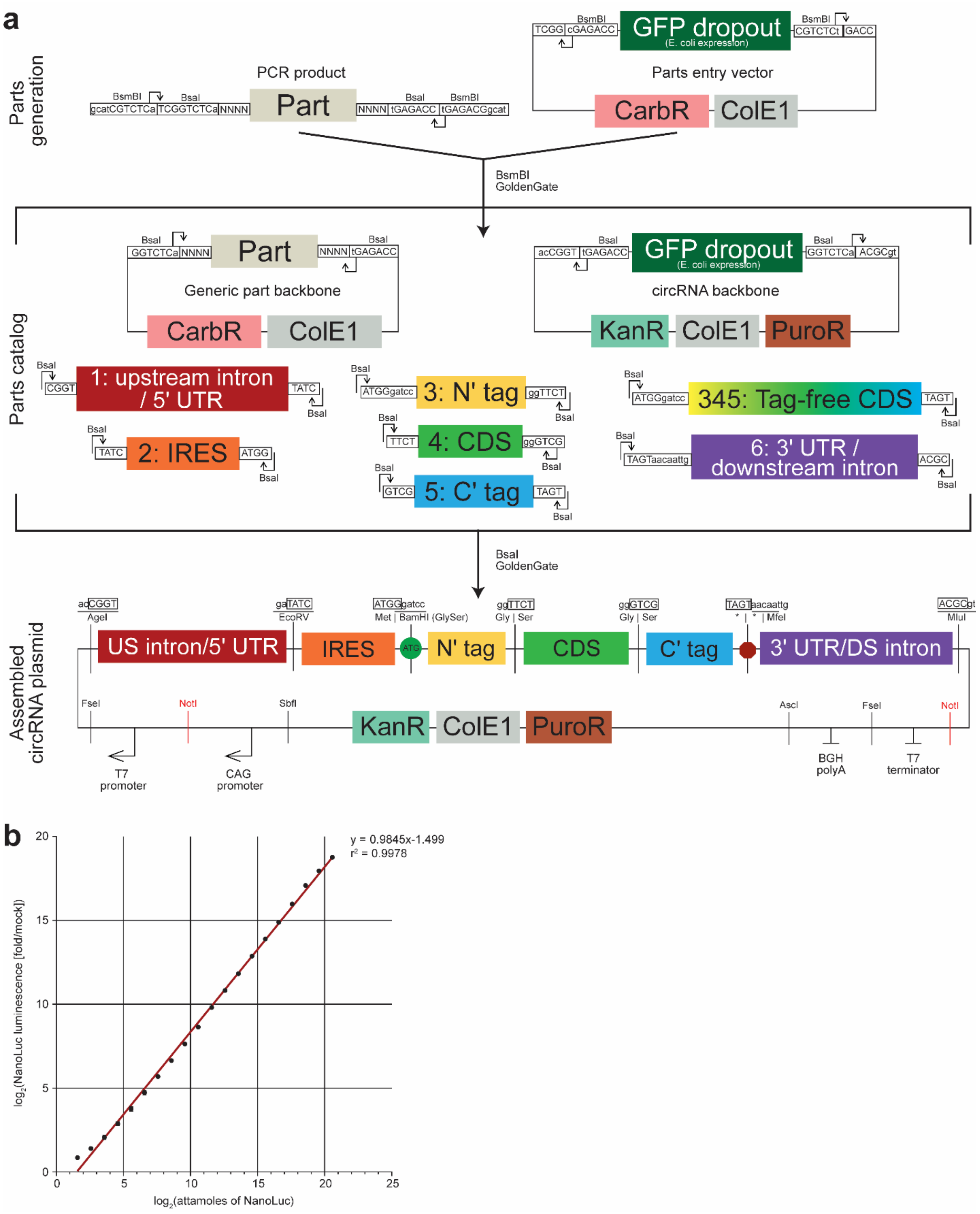

Supplementary Figure 1. Details of modular cloning platform and NanoLuc assay.

a, Detailed schematic describing the modular cloning platform used to create template plasmids for circRNA synthesis. Part plasmids containing parts 1-6 flanked by the indicated overhangs

were synthesized by combining a PCR product or premade DNA fragment with the parts entry vector in a BsmBI Golden Gate reaction. These parts corresponded to the upstream intron and 5' untranslated region (UTR), IRES, N-terminal (N') tag, coding sequence (CDS), C-terminal (C') tag, and 3' UTR and downstream intron. Part plasmids and the circRNA backbone were subsequently combined in a BsaI Golden Gate reaction to create a circRNA plasmid. Fully assembled circRNA plasmids included the indicated restriction enzyme cut sites at key junctions to facilitate subcloning and in-frame glycine-serine linkers between parts 3, 4, and 5. Linkers were omitted if parts 3-5 were replaced with a single part.

b, Standard curve quantification of the NanoLuc assay used to assess circRNA translation, demonstrating linearity across a 2^{21} (2 million)-fold dilution range. Data are mean for n=3 technical replicates.

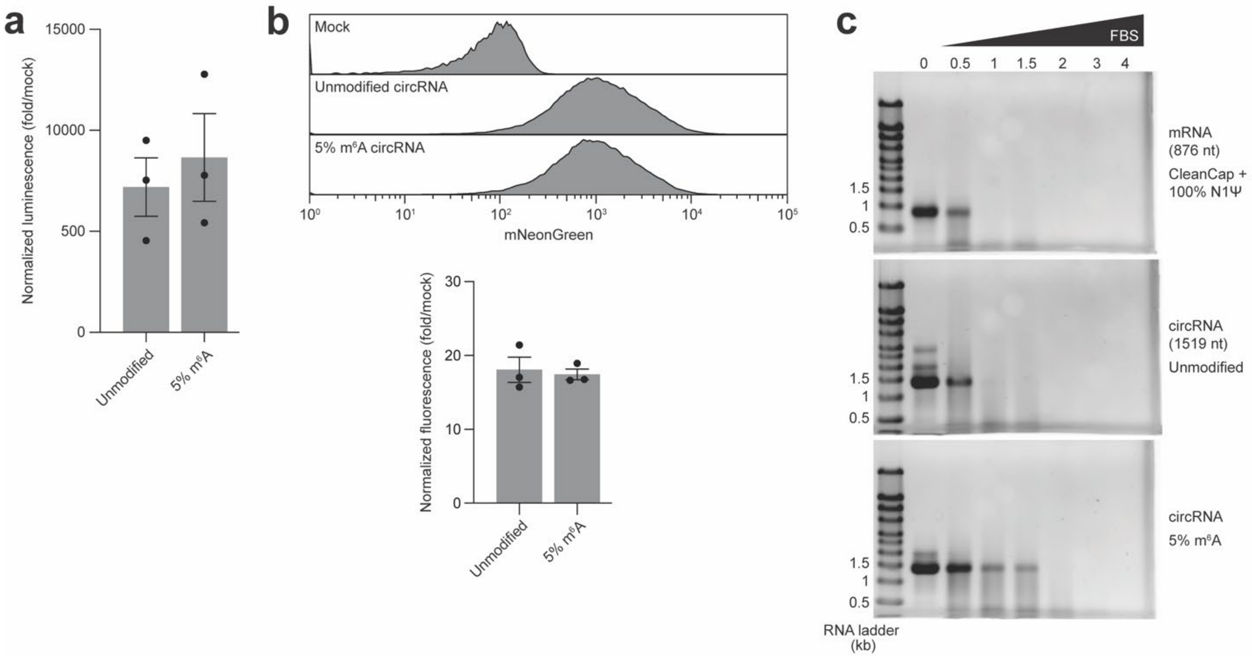

Supplementary Figure 2. m^{6}A incorporation preserves circRNA translation and improves resistance to degradation.

a, NanoLuc activity after transfection of HeLa cells with unmodified circRNA or circRNA containing 5% m^{6}A. NanoLuc activity was normalized to constitutive firefly luciferase activity from the same sample, then divided by values from mock transfection. Data are mean ± SEM for n=3 biological replicates.

b, mNeonGreen fluorescence at 24 hours after electroporation of HeLa cells with unmodified circRNA or circRNA containing 5% m^{6}A. Mean mNeonGreen expression was measured by flow cytometry and normalized by values from mock electroporation. Data are histograms for n>50,000 live singlet cells per condition and mean ± SEM for n=3 biological replicates.

c, Resistance of mRNA and circRNAs to degradation in escalating doses of fetal bovine serum (FBS). RNAs were incubated in the indicated percentages of FBS at 37°C for 30 minutes, then briefly denatured in RNA loading buffer before gel electrophoresis. The same amount of ladder per gel and RNA per well were used to allow for comparisons between gels. Data are from one of two experiments with similar results.

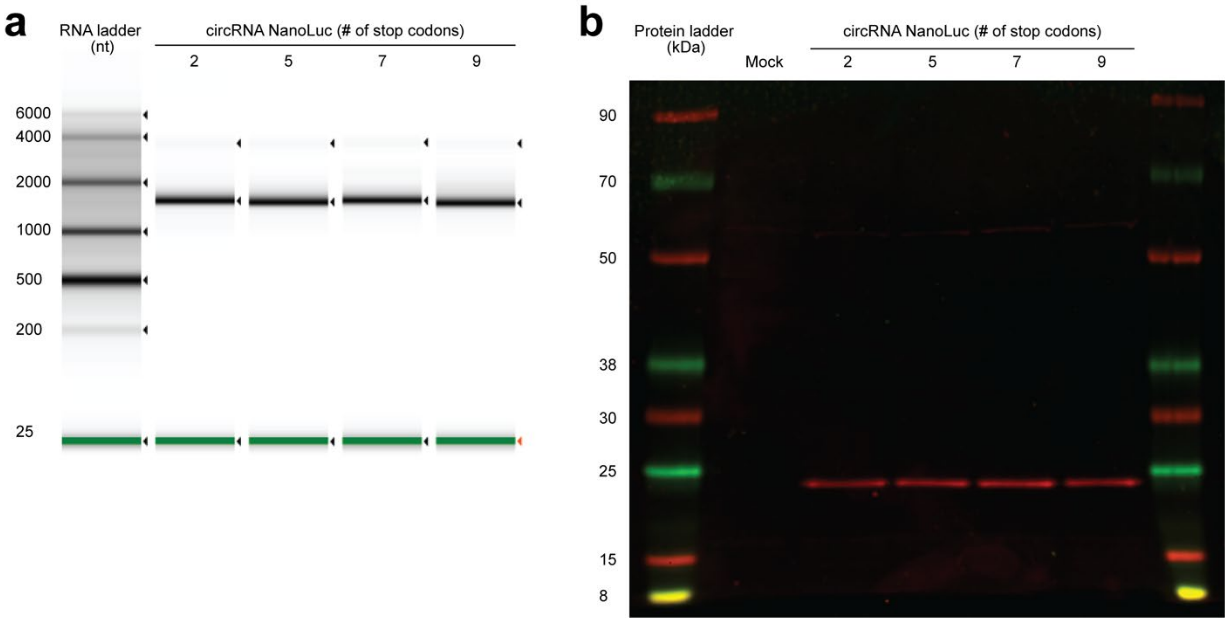

Supplementary Figure 3. Additional stop codons do not change circRNA or protein size.

a, TapeStation gel electrophoresis depicting the size of circRNAs encoding NanoLuc and possessing the indicated number of stop codons. Data are from one of at least two experiments with similar results.

b, Western blot depicting NanoLuc protein in HeLa lysate at 24 hours after electroporation with circRNAs encoding NanoLuc and possessing the indicated number of stop codons. Each lane was loaded with 10 µg of total protein. Data are from one of two experiments with similar results.

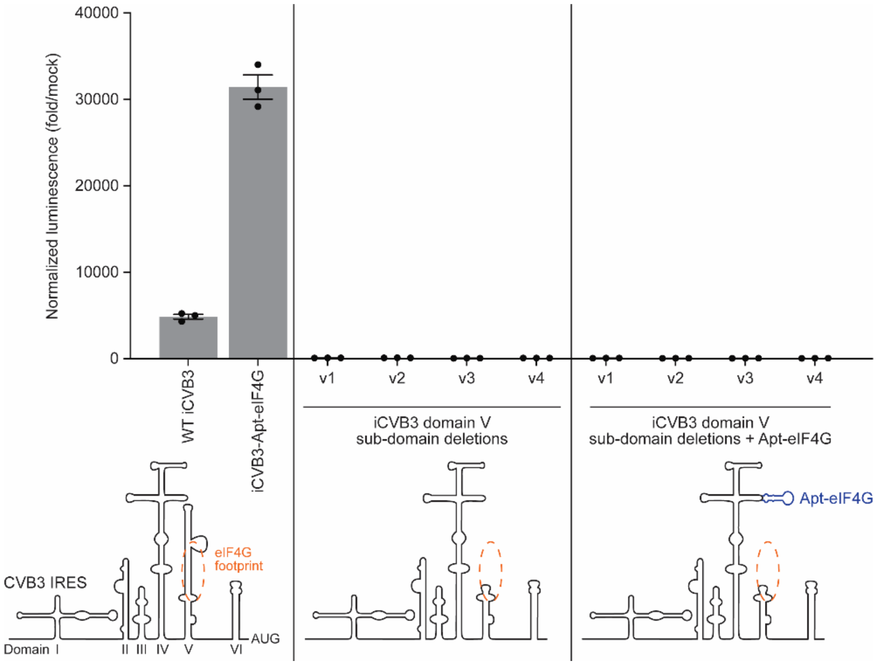

Supplementary Figure 4. eIF4G-binding site deletions are translation-lethal and irrecoverable.

NanoLuc activity at 24 hours after transfection of HeLa cells with circRNAs containing wild-type iCVB3, iCVB3 with Apt-eIF4G insertion, iCVB3 with domain V sub-domain deletions, or iCVB3 with domain V sub-domain deletions and attempted rescue with Apt-eIF4G. Sub-domain deletions (v1-v4) differed in the position where the stem loop was truncated, but at a minimum all ablated the eIF4G footprint. NanoLuc activity was normalized to constitutive firefly luciferase activity from the same sample, then divided by values from mock transfection. Data are mean ± SEM for n=3 biological replicates.

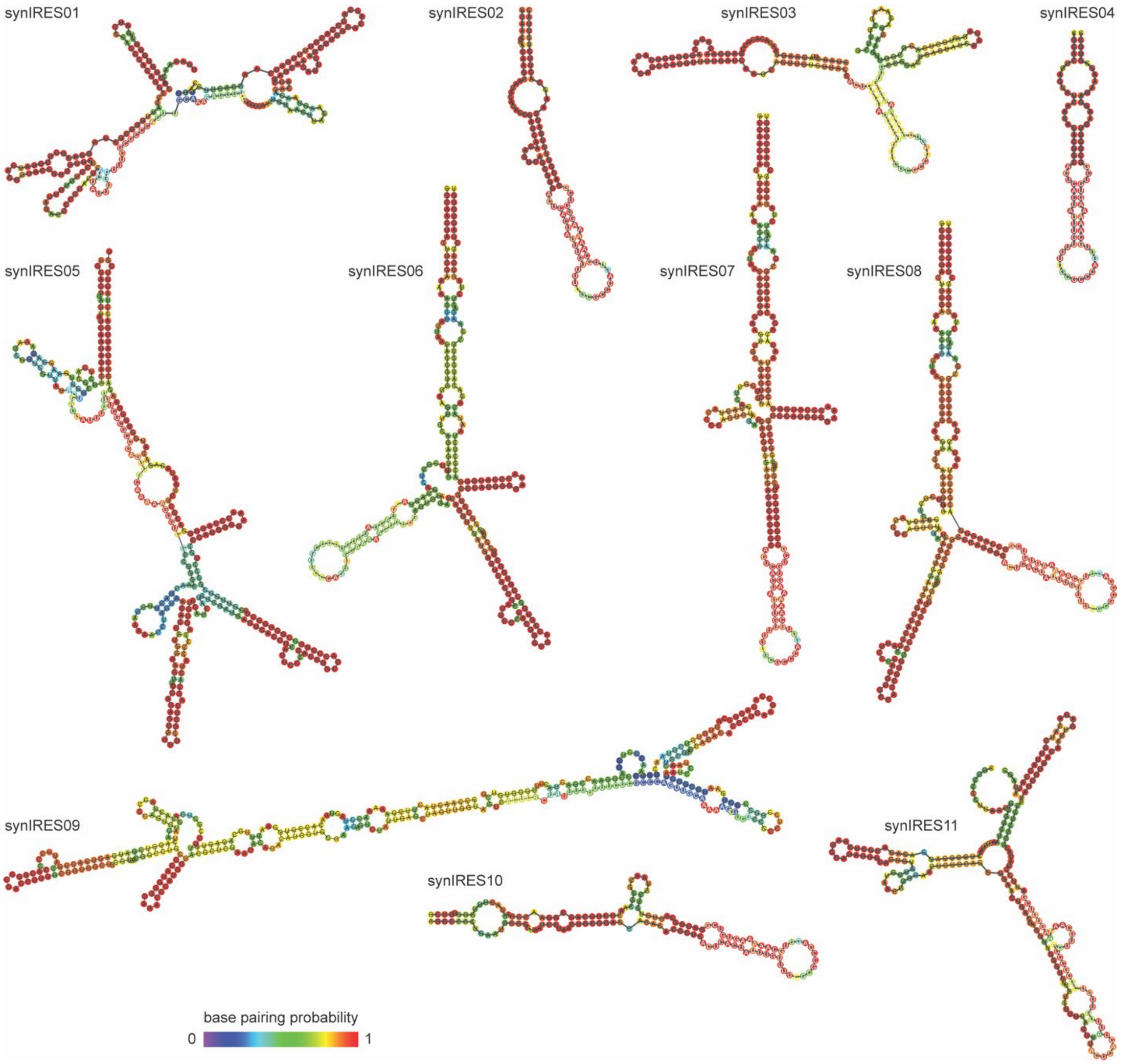

Supplementary Figure 5. *In silico* RNA structure prediction can inform IRES engineering.

RNA structure predictions for synthetic IRESs synIRES01-11 at the site of aptamer insertion. For inter-domain insertions (synIRES01, 03, 05, 09, and 11), structure prediction was performed on Apt-eIF4G and the adjacent iCVB3 domains. For loop insertions (synIRES02, 04, 06, 07, 08, and 10), structure prediction was performed on Apt-eIF4G and the iCVB3 domain containing the insertion. In each structure, nucleotides corresponding to Apt-eIF4G are shown in white.

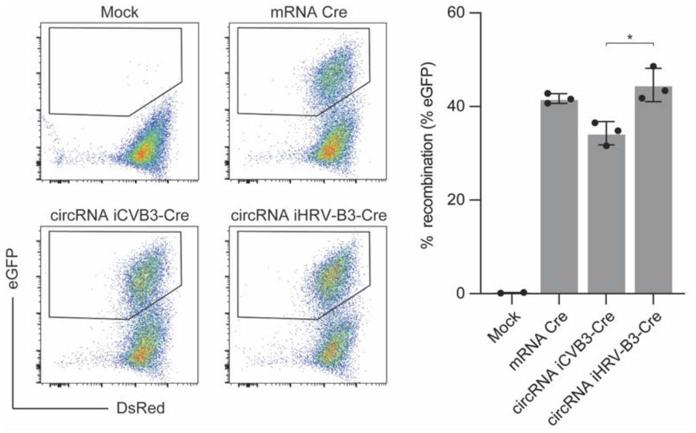

Supplementary Figure 6. iHRV-B3 enhances the activity of circRNA encoding Cre.

Percentage of eGFP-positive cells at 24 hours after transfection of CreR-293T cells with mRNA or circRNAs encoding Cre recombinase. In this cell line, DsRed is expressed before Cre recombination and eGFP is expressed after. eGFP and DsRed expression were measured by flow cytometry. Data are mean ± SEM for n=2 biological replicates for mock, n=3 biological replicates for all other conditions. * *P*=0.0147 by unpaired two-sided t-test.

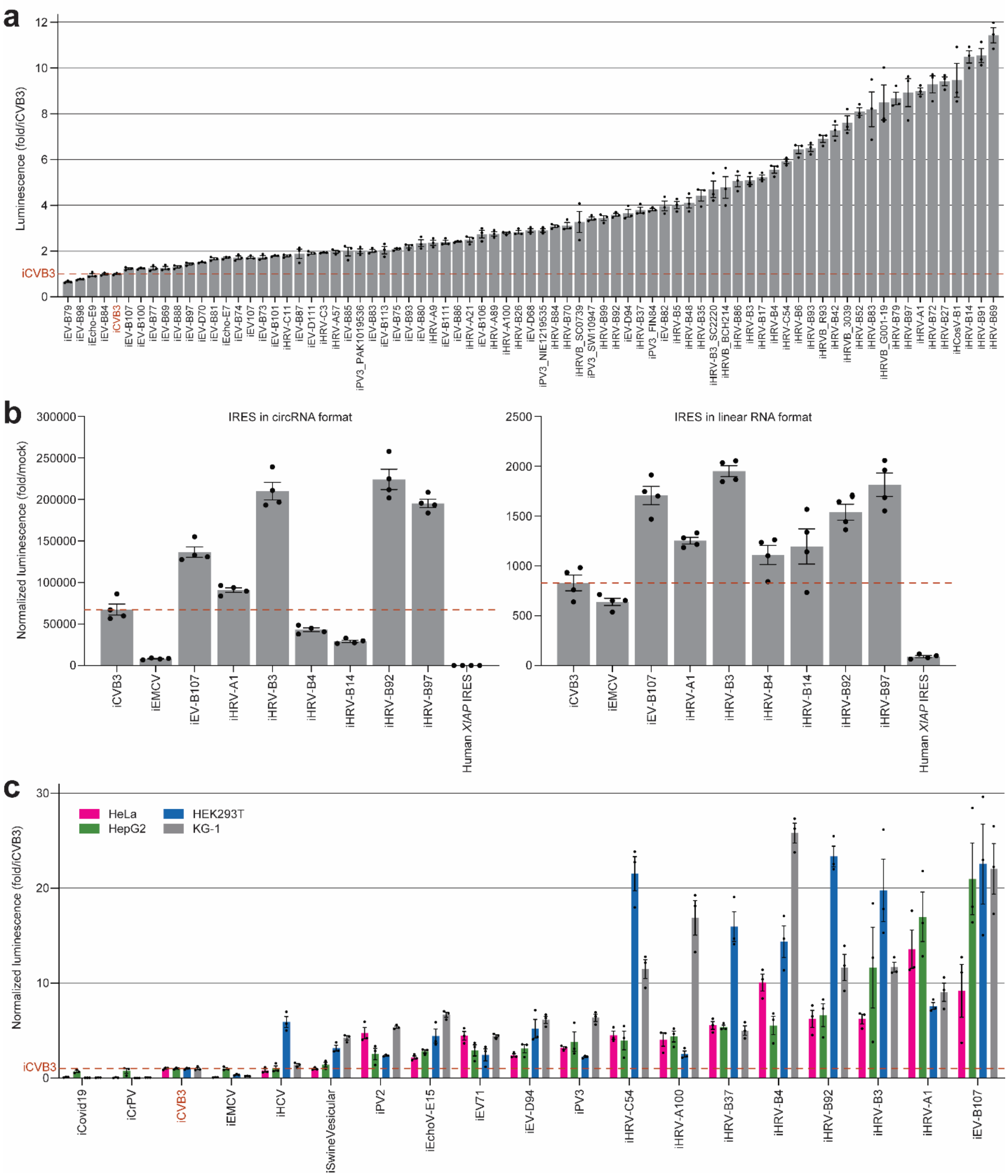

Supplementary Figure 7. Subsets of enterovirus and human rhinovirus B IRESs drive strong circRNA translation.

a, NanoLuc activity after *in vitro* transcription-translation (IVTT) of circRNA plasmids containing enterovirus (EV) or human rhinovirus B (HRV-B) IRESs. All known EV and HRV-B IRES sequences were cloned into circRNA plasmids. Purified plasmids were then subjected to IVTT

using HeLa lysate. NanoLuc activity was divided by values from mock IVTT. Data are mean ± SEM for n=4 biological replicates.

b, NanoLuc activity at 24 hours after transfection of HeLa cells with circRNAs or linear RNAs containing strong IRESs from the IVTT-based screen. Linear RNA sequences were identical to those of circRNAs with the exclusion of self-splicing introns. NanoLuc activity was normalized to constitutive firefly luciferase activity from the same sample, then divided by values from mock transfection. Data are mean ± SEM for n=4 biological replicates.

c, NanoLuc activity at 24 hours after transfection of HeLa, HepG2, HEK293T, and KG-1 cells with circRNAs containing the indicated IRESs. Values for HeLa, HepG2, and HEK293T cells are the same as in Fig. 5a. NanoLuc activity was normalized to constitutive firefly luciferase activity from the same sample, then divided by values from transfection with circRNA containing iCVB3. Data are mean ± SEM for n=3 biological replicates.

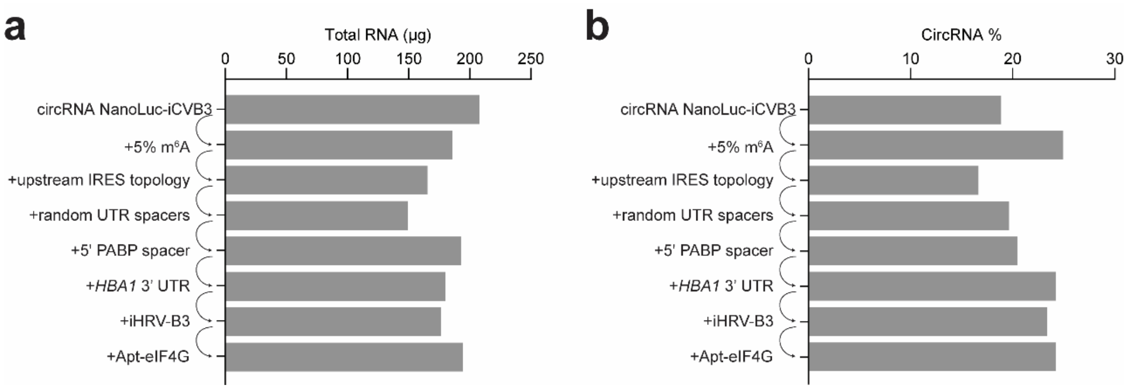

Supplementary Figure 8. CircRNA optimizations do not adversely affect circRNA synthesis.

a, Effect of successive circRNA optimizations on total RNA production when starting from 1 µg of *in vitro* transcription template.

b, Effect of successive circRNA optimizations on circRNA percentage, defined as the percentage of RNA remaining after digestion with RNaseR.

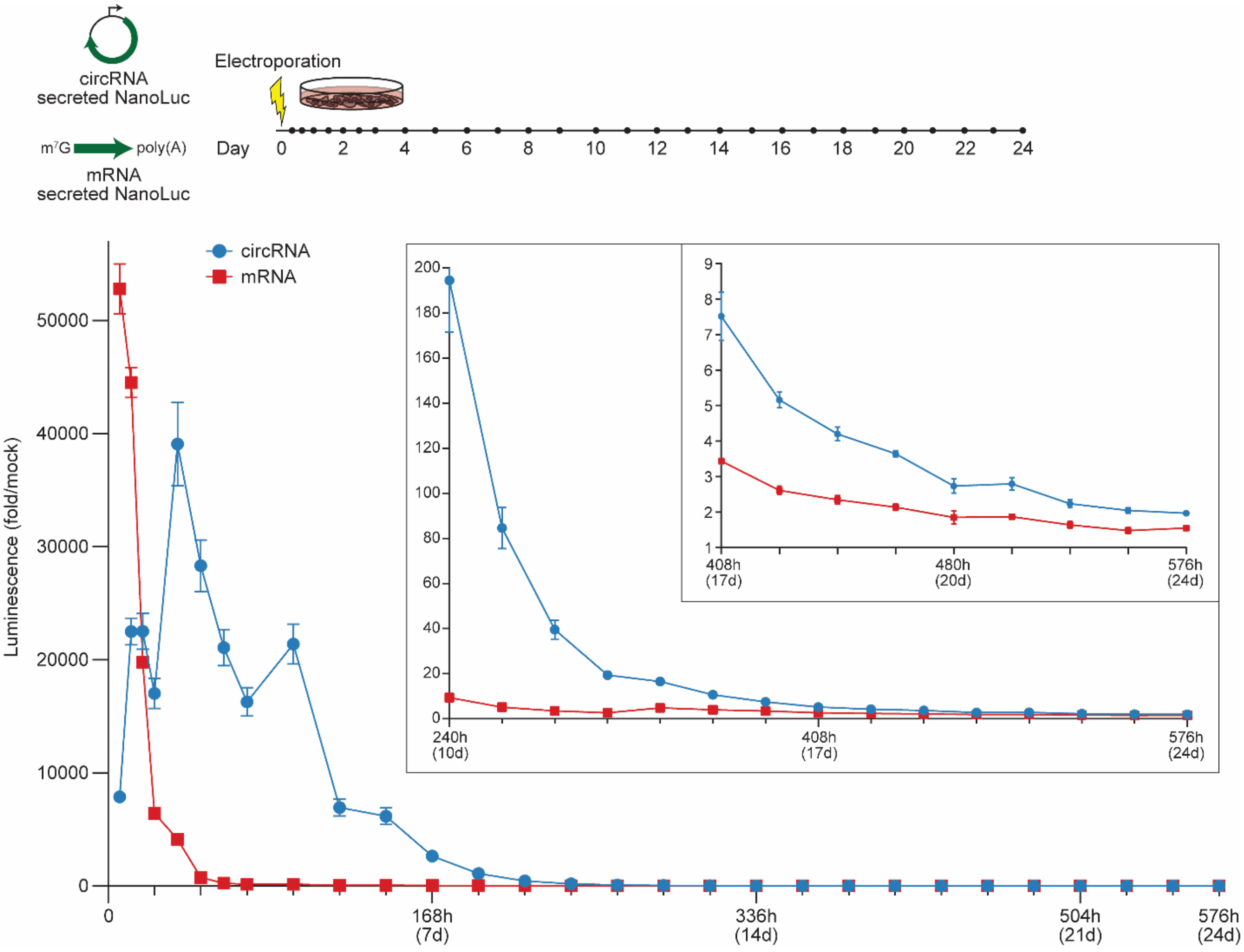

Supplementary Figure 9. CircRNA exhibits more durable translation than mRNA *in vitro.*

NanoLuc activity in supernatant after electroporation of HeLa cells with circRNA or mRNA encoding secreted NanoLuc. CircRNA was synthesized with 5% m^6A incorporation and the HRV-B3 IRES. mRNA was synthesized with CleanCap reagent, 100% N1Ψ incorporation, and a 120 nt poly(A) tail. At the indicated hours (h) and days (d) post-electroporation, media was harvested to assay secreted NanoLuc and replaced. NanoLuc activity was divided by values from mock electroporation. Data are mean ± SEM for n=3 biological replicates.

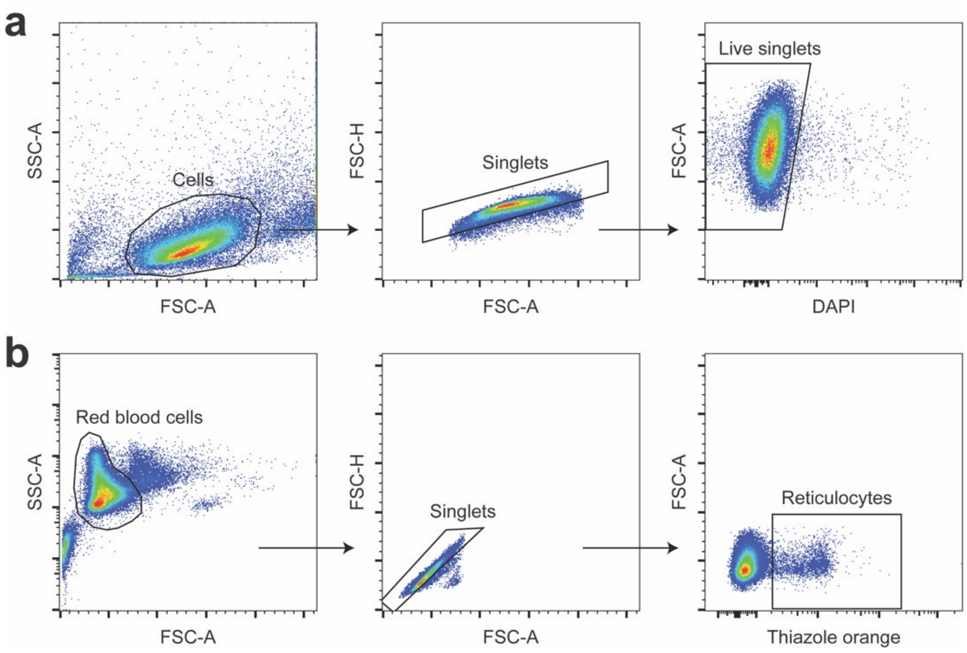

Supplementary Figure 10. Flow cytometry gating.

a, Gating strategy to analyze live singlet HEK293T cells after electroporation.

b, Gating strategy to analyze reticulocytes from peripheral mouse blood.

SUPPLEMENTARY METHODS

RNA gel electrophoresis

1% agarose gels were prepared by melting RNase-free agarose in Tris-acetate-EDTA running buffer with addition of ethidium bromide. RNA was denatured in RNA loading buffer (Thermo Fisher) by diluting 1:1 volumetrically, heating at 72°C for 3 minutes, and cooling on ice for 1 minute. RNA was loaded into each well and run at 100 V at room temperature. Images were taken using a Bio-Rad Gel Doc XR and Image Lab 5.2 software using the "SYBR-Safe" settings.

Western blotting

HeLa cells were lysed 24 hours after electroporation using RIPA Lysis and Extraction Buffer (Thermo Fisher) containing Halt Protease and Phosphatase Inhibitor Cocktail (Thermo Fisher). The resulting lysates were clarified by centrifugation and quantified for protein using bicinchoninic acid. 10 μg of total protein from each sample was separated on a Bis-Tris gel and transferred to a nitrocellulose membrane using the iBlot 2 Gel Transfer Device. After blocking with 5% bovine serum albumin in 0.1% Tween-20 diluted in PBS for one hour at room temperature, the membrane was stained with a 1:500 dilution of anti-NanoLuc antibody (R&D Systems, MAB10026) in blocking buffer overnight at 4°C. Following washes, the membrane was then incubated with a 1:10,000 dilution of IRDye 680RD goat anti-mouse secondary antibody (LI-COR Biosciences, 926-68070) and visualized on an Odyssey CLx Imaging System (LI-COR Biosciences) using Image Studio 3.1 software.

RNA structure predictions

RNA structures were predicted using the RNAfold web server (http://rna.tbi.univie.ac.at/cgi-bin/RNAWebSuite/RNAfold.cgi) with default settings except for

deselecting “avoid isolated base pairs.” The optimal secondary structure based on minimal free energy prediction was subsequently used to represent the RNA sequence.

Cre reporter assay

Cre reporter loxP-DsRED-STOP-loxP-eGFP (Addgene, #62732) was used to create a lentiviral derived stable Cre reporter HEK293T cell line (CreR-293T). For virus production, HEK293T cells were co-transfected with pMD2.G (Addgene, #12259), psPAX2 (Addgene, #12260), and a plasmid encoding the Cre reporter lentivirus using Lipofectamine 3000 (Thermo Fisher) following manufacturer’s instructions. The supernatant containing viral particles was collected 48 hours after transfection, concentrated using Lenti-X concentrator (Clontech), and stored at -80°C. Viral particles were added to 25% confluent cells at a multiplicity of infection of 1. Cre reporter expressing HEK293T cells were selected 48 hours after transduction using puromycin (Thermo Fisher) a final concentration of 1 µg/mL, maintained in selection media for seven days, and allowed to recover for one day before downstream experiments.

CircRNA and mRNA encoding Cre recombinase were synthesized as described above using the Cre sequence from LV-Cre pLKo.1 plasmid (Addgene, #25997). Stable CreR-293T cells were seeded in a 12-well plate at 300,000 cells per well and transfected with 4 µg of circRNA or mRNA encoding Cre using Lipofectamine 2000 (Thermo Fisher). Cre-mediated recombination was analyzed 24 hours after transfection on an Attune NxT flow cytometer.
