# Supplementary material for: Engineering circular RNA for enhanced protein production
Source: Nat Biotechnol. 2022 Jul 18;41(2):262–72. doi: 10.1038/s41587-022-01393-0 (PMC9931579; doi:10.1038/s41587-022-01393-0)
Supplement: Supplementary file 2 — Reporting Summary [file 41587_2022_1393_MOESM2_ESM.pdf]

nature research

Corresponding author(s): Howard Y. Chang

Last updated by author(s): 6/8/2022

Reporting Summary

Nature Research wishes to improve the reproducibility of the work that we publish. This form provides structure for consistency and transparency in reporting. For further information on Nature Research policies, see our Editorial Policies and the Editorial Policy Checklist.

Statistics

For all statistical analyses, confirm that the following items are present in the figure legend, table legend, main text, or Methods section.

n/a	Confirmed	
☐	☒	The exact sample size (*n*) for each experimental group/condition, given as a discrete number and unit of measurement
☐	☒	A statement on whether measurements were taken from distinct samples or whether the same sample was measured repeatedly
☐	☒	The statistical test(s) used AND whether they are one- or two-sided<br>*Only common tests should be described solely by name; describe more complex techniques in the Methods section.*
☒	☐	A description of all covariates tested
☐	☒	A description of any assumptions or corrections, such as tests of normality and adjustment for multiple comparisons
☐	☒	A full description of the statistical parameters including central tendency (e.g. means) or other basic estimates (e.g. regression coefficient) AND variation (e.g. standard deviation) or associated estimates of uncertainty (e.g. confidence intervals)
☐	☒	For null hypothesis testing, the test statistic (e.g. *F*, *t*, *r*) with confidence intervals, effect sizes, degrees of freedom and *P* value noted<br>*Give P values as exact values whenever suitable.*
☒	☐	For Bayesian analysis, information on the choice of priors and Markov chain Monte Carlo settings
☒	☐	For hierarchical and complex designs, identification of the appropriate level for tests and full reporting of outcomes
☒	☐	Estimates of effect sizes (e.g. Cohen's *d*, Pearson's *r*), indicating how they were calculated

Our web collection on statistics for biologists contains articles on many of the points above.

Software and code

Policy information about availability of computer code

Data collection	No code was generated by this study. Software used to collect data include i-control 1.10, SoftMax Pro 7.1, Image Lab 5.2, and Image Studio 3.1. RNA structures were predicted using the RNAfold web server (http://rna.tbi.univie.ac.at/cgi-bin/RNAWebSuite/RNAfold.cgi).
Data analysis	No code was generated by this study. Software used to analyze data include Microsoft Excel 16, Prism 9, Flowjo 10, and Aura 4.0.

For manuscripts utilizing custom algorithms or software that are central to the research but not yet described in published literature, software must be made available to editors and reviewers. We strongly encourage code deposition in a community repository (e.g. GitHub). See the Nature Research guidelines for submitting code & software for further information.

Data

Policy information about availability of data

All manuscripts must include a data availability statement. This statement should provide the following information, where applicable:
- Accession codes, unique identifiers, or web links for publicly available datasets
- A list of figures that have associated raw data
- A description of any restrictions on data availability

Source data for figures are provided in the manuscript.

Field-specific reporting

Please select the one below that is the best fit for your research. If you are not sure, read the appropriate sections before making your selection.

☒ Life sciences ☐ Behavioural & social sciences ☐ Ecological, evolutionary & environmental sciences

For a reference copy of the document with all sections, see nature.com/documents/nr-reporting-summary-flat.pdf

Life sciences study design

All studies must disclose on these points even when the disclosure is negative.

Sample size	3-4 biological replicates were used per condition in all experiments to allow for statistical analyses while enabling all samples within an experiment to be concurrently processed. This sample size was sufficient to capture larger statistically significant differences between groups
Data exclusions	3 data points from Figure 5a were excluded due to a pipetting error. Otherwise, no data were excluded from analyses
Replication	Findings were replicated at least twice - at least once in pilot experiments as well as once in the presented experiments
Randomization	Samples and mice were randomly assigned to experimental groups
Blinding	Investigators were not blinded to group allocations due to personnel constraints

Reporting for specific materials, systems and methods

We require information from authors about some types of materials, experimental systems and methods used in many studies. Here, indicate whether each material, system or method listed is relevant to your study. If you are not sure if a list item applies to your research, read the appropriate section before selecting a response.

Materials & experimental systems

n/a	Involved in the study	
☐	☒	Antibodies
☐	☒	Eukaryotic cell lines
☒	☐	Palaeontology and archaeology
☐	☒	Animals and other organisms
☒	☐	Human research participants
☒	☐	Clinical data
☒	☐	Dual use research of concern

Methods

n/a	Involved in the study	
☒	☐	ChIP-seq
☐	☒	Flow cytometry
☒	☐	MRI-based neuroimaging

Antibodies

Antibodies used	Anti-NanoLuc antibody (R&D Systems, MAB10026), IRDye 680RD goat anti-mouse secondary antibody (LI-COR Biosciences, 926-68070)
Validation	Antibodies were validated per manufacturers and were not subjected to validation experiments in this study

Eukaryotic cell lines

Policy information about cell lines

Cell line source(s)	HeLa (CCL-2), HEK293T (CRL-11268), HepG2 (HB-8065), and KG-1 (CCL-246) cell lines were obtained from ATCC
Authentication	Cell lines were not authenticated
Mycoplasma contamination	Cell lines were not tested for mycoplasma contamination
Commonly misidentified lines (See ICLAC register)	No commonly misidentified lines were used

Animals and other organisms

Policy information about studies involving animals; ARRIVE guidelines recommended for reporting animal research

Laboratory animals	All animal experiments were performed in 2-6 month old female BALB/c mice obtained from The Jackson Laboratory. Animals were housed at room temperature in humidity-controlled rooms with a 12-hour light/dark cycle.
Wild animals	This study did not involve wild animals
Field-collected samples	This study did not involve field-collected samples
Ethics oversight	All experimental procedures were approved by the Institutional Animal Care and Use Committee at Stanford University

Note that full information on the approval of the study protocol must also be provided in the manuscript.

Flow Cytometry

Plots

Confirm that:

☒ The axis labels state the marker and fluorochrome used (e.g. CD4-FITC).

☒ The axis scales are clearly visible. Include numbers along axes only for bottom left plot of group (a 'group' is an analysis of identical markers).

☒ All plots are contour plots with outliers or pseudocolor plots.

☒ A numerical value for number of cells or percentage (with statistics) is provided.

Methodology

Sample preparation	For mNeonGreen analysis, cells were lifted using warmed TrypLE, which was quenched with DMEM, and incubated in PBS containing DAPI live-dead stain. For reticulocyte analysis, 10 µL of blood was collected from the tail vein of each mouse and incubated with 1 mL of Reticulocyte Reagent System reagent following manufacturer's instructions.
Instrument	For mNeonGreen analysis, flow cytometry was performed using an Attune NxT flow cytometer. For reticulocyte analysis, flow cytometry was performed on a BD LSR II flow cytometer.
Software	Data was analyzed using FlowJo 10
Cell population abundance	For mNeonGreen analysis, flow cytometry was performed on bulk cells. For reticulocyte analysis, abundances are displayed in Figure 6g as a percentage of erythrocytes, which constituted the vast majority of cells.
Gating strategy	See Supplementary Fig. 10. For mNeonGreen analysis, cells were gated using FSC/SSC to exclude debris and doublets and for viability using DAPI. For reticulocyte analysis, erythrocytes were gated using FSC/SSC to exclude debris, doublets, and larger leukocytes, and then gated for Reticulocyte Reagent System reagent positivity.

☒ Tick this box to confirm that a figure exemplifying the gating strategy is provided in the Supplementary Information.
